# Resilience and Vulnerability to Stress-Induced Anhedonia: Unveiling Brain Gene Expression and Mitochondrial Dynamics in a Mouse Chronic Stress Depression Model

**DOI:** 10.3390/biom13121782

**Published:** 2023-12-12

**Authors:** Tatyana Strekalova, Evgeniy Svirin, Anna Gorlova, Elizaveta Sheveleva, Alisa Burova, Adel Khairetdinova, Kseniia Sitdikova, Elena Zakharova, Alexander M. Dudchenko, Aleksey Lyundup, Sergey Morozov

**Affiliations:** 1 Division of Molecular Psychiatry, Center of Mental Health, University of Hospital Würzburg, 97080 Wuerzburg, Germany; 2 Institute of General Pathology and Pathophysiology, Russian Academy of Medical Sciences, Moscow 125315, Russiaanna.gorlova204@gmail.com (A.G.); shevelevalisa02@gmail.com (E.S.); burova.ae@phystech.edu (A.B.); khairetdinova.adele@gmail.com (A.K.); sitdikova.kk@gmail.com (K.S.); zakharova.ei.27@gmail.com (E.Z.); amdudchenko@gmail.com (A.M.D.); sergey_moroz@list.ru (S.M.); 3 Endocrinology Research Centre, Dmitry Ulyanov St. 19, Moscow 117036, Russia; lyundup2020@gmail.com; 4 Research and Education Resource Center, Peoples Friendship University of Russia (RUDN University), 6 Miklukho-Maklaya St, Moscow 117198, Russia

**Keywords:** major depression, cognitive deficits, metabolic regulation, insulin receptor sensitizer, hippocampus, anhedonia, chronic stress, stress resilience, mouse

## Abstract

The role of altered brain mitochondrial regulation in psychiatric pathologies, including Major Depressive Disorder (MDD), has attracted increasing attention. Aberrant mitochondrial functions were suggested to underlie distinct inter-individual vulnerability to stress-related MDD syndrome. In this context, insulin receptor sensitizers (IRSs) that regulate brain metabolism have become a focus of recent research, as their use in pre-clinical studies can help to elucidate the role of mitochondrial dynamics in this disorder and contribute to the development of new antidepressant treatment. Here, following 2-week chronic mild stress (CMS) using predation, social defeat, and restraint, MDD-related behaviour and brain molecular markers have been investigated along with the hippocampus-dependent performance and emotionality in mice that received the IRS dicholine succinate (DS). In a sucrose test, mice were studied for the key feature of MDD, a decreased sensitivity to reward, called anhedonia. Based on this test, animals were assigned to anhedonic and resilient-to-stress-induced-anhedonia groups, using a previously established criterion of a decrease in sucrose preference below 65%. Such assignment was based on the fact that none of control, non-stressed animals displayed sucrose preference that would be smaller than this value. DS-treated stressed mice displayed ameliorated behaviours in a battery of assays: sucrose preference, coat state, the Y-maze, the marble test, tail suspension, and nest building. CMS-vulnerable mice exhibited overexpression of the inflammatory markers *Il-1β*, *tnf*, and *Cox-1*, as well as *5-htt* and *5-ht2a*-R, in various brain regions. The alterations in hippocampal gene expression were the closest to clinical findings and were studied further. DS-treated, stressed mice showed normalised hippocampal expression of the plasticity markers *Camk4*, *Camk2*, *Pka*, *Adcy1*, *Creb-ar*, *Nmda-2r-ar*, and *Nmda-2r-s*. DS-treated and non-treated stressed mice who were resilient or vulnerable to anhedonia were compared for hippocampal mitochondrial pathway regulation using Illumina profiling. Resilient mice revealed overexpression of the mitochondrial complexes NADH dehydrogenase, succinate dehydrogenase, cytochrome *bc*1, cytochrome *c* oxidase, F-type and V-type ATPases, and inorganic pyrophosphatase, which were decreased in anhedonic mice. DS partially normalised the expression of both ATPases. We conclude that hippocampal reduction in ATP synthesis is associated with anhedonia and pro-inflammatory brain changes that are ameliorated by DS.

## 1. Introduction

Growing evidence has related altered brain metabolism to the key symptoms of Major Depressive Disorder (MDD) and conditions associated with this disease [1,2], as well as stress-related pathologies [3,4,5] and other neuropsychiatric conditions sharing clinical features with MDD [6,7,8,9,10]. Conversely, early studies have shown that 70% of patients diagnosed with mitochondrial disorders met the diagnostic criteria for mental disorders, including depression and cognitive problems [11]. It has been established that sufficient mitochondrial abundance and function are important prerequisites for synapse formation and remodelling [12,13], neuronal survival/neuroprotective processes [14,15], and synaptic plasticity [12,13], including hippocampal long-term potentiation (LTP) and depression (LTD), learning, and memory [16,17].

Numerous studies have established that insulin receptor (IR)-mediated signalling modulates mitochondrial functions [18,19,20]. Abnormalities in IR-mediated signalling are associated with neuropsychiatric disorders, such as MDD [21,22,23,24,25], autism [26], schizophrenia [22], and anxiety disorders [3,27]. IR sensitizers (IRSs) potentiate the binding of insulin to its receptor via various mechanisms that enable insulin efficacy, even at sub-threshold concentrations; as such, these compounds have been called ‘sensitizers of the neuronal insulin receptor’ [28]. There is growing evidence suggesting that enhanced IR signalling with IR sensitizers (IRSs) can be beneficial for patients with these pathologies [20]. For example, an IRS can be used effectively as add-on treatment for patients with depression, particularly when MDD is co-morbid with metabolic syndrome [29,30].

Previous publications have demonstrated antidepressant-like effects, increased neuronal mitochondrial biogenesis, decreased neuronal damage, and anti-inflammatory properties for various IRSs [31,32,33]. For example, clinical and pre-clinical studies have shown that thiazolidinediones exert an antidepressant effect [34,35,36,37,38,39,40,41,42]. Specifically, the thiazolidinediones rosiglitazone and pioglitazone induce a therapeutic effect in patients with MDD who are refractory to standard antidepressant treatment and have insulin resistance [38,39,43,44]. Antidepressant-like properties have also been reported for a mitochondrial complex II substrate, Dicholine Succinate (DS), in various rodent models of stress and MDD [45,46,47,48]. DS dose-dependently stimulates insulin-dependent H2O2 synthesis of the mitochondrial respiratory chain in neuronal culture, thus enhancing the IR function via insulin-stimulated autophosphorylation of IR kinase at tyrosine residues in neurons, which is a trigger for the activation of IR [18,31,49].

In this work, we have used DS to investigate the role of mitochondrial metabolism in the MDD-like state in a pre-clinical model of this disorder. Because MDD is a psychiatric disease for which the currently available treatment is far from sufficiently effective—as only up to 70% of patients respond to standard antidepressants [50]—the need to develop new MDD therapies remains high. Up until now, the link between compromised brain metabolism and symptoms of MDD has mostly been shown in clinical studies [51,52], which unfortunately do not quite allow for dissecting the specific molecular mitochondrial mechanisms underlying aberrant brain metabolic changes associated with MDD. Recent studies have revealed candidate genes encoding mitochondria-related proteins and alterations in the mitochondrial genome. These findings suggest that endophenotypes of MDD, where the mitochondrial function is impaired, may be the leading cause of depressive symptomatology, and they may accompany cognitive dysfunction [52].

A variety of MDD endophenotypes may share a common basis with the general phenomenon of inter-individual variability in response to environmental challenges that have been studied more frequently in pre-clinical models of depression [53,54,55,56,57]. Some of these studies have revealed differential metabolic mitochondrial changes in animals that display distinct vulnerability to stress-induced MDD, as has been shown for the prefrontal cortex (PFC) of mice exposed to chronic restraint stress [58]. Researchers have argued that the hippocampus is the structure that plays an important role in governing an individual’s susceptibility or resilience to stress-induced depression [59,60,61,62]. Concurrently, several molecular and cellular mechanisms constituting the biological basis of these phenomena have been described for the hippocampus [63,64]. Hence, we have aimed to study metabolic and molecular hippocampal changes in the context of individual differences in vulnerability versus resilience to the MDD-like syndrome in experimental animals.

Anhedonia, decreased sensitivity to a reward, is considered a key feature of MDD [65,66]. Unlike another core symptom of this disorder, depressed mood [9,10,67], it can be induced and measured in small rodents [67,68]. In MDD patients, hedonic deficit was previously established to be associated with cognitive symptoms [41,42], impaired plasticity in the hippocampus [67,68], and other key neurochemical and cellular correlates of MDD [67,68]. Therefore, the use of animal models that represent Recapitulate anhedonia can be advantageous over other approaches in translational research of the biological basis of depression.

In this study, we have applied a variant of the chronic mild stress (CMS) paradigm, which is based on the induction of anhedonia [67,68]. With the employed stress protocol, 50–70% of stressed mice were previously described as displaying individual vulnerability to hedonic deficit [47,69,70,71]. The anhedonic state in stressed mice is defined by a decrease in sucrose preference below the lowest values shown by non-stressed controls, i.e., 65% [47,69,70,71]. Consequently, the non-anhedonic, resilient animals can be considered an internal control for the effects of stress that are not associated with MDD-like changes. Therefore, the correlates of the depressive-like syndrome can be separated from the effects of stress that are not associated with this syndrome, and they can be investigated with improved accuracy [57,71,72].

**Figure 1:**
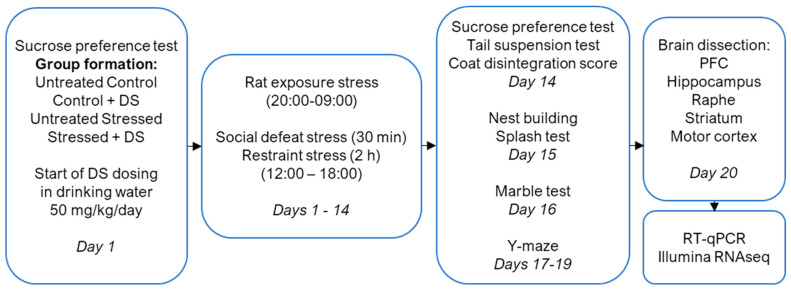
Experiment design. A chronic mild stress study was carried out on mice (*n* = 33) that were either untreated or received a pharmacological intervention with DS. On day 1, mice were tested in the sucrose preference test and assigned to stress group and control groups: control untreated, control DS-treated, stressed untreated, and stressed DS-treated mice. On the same day, the administration of DS was started with drinking water at a dose of 50 mg/kg/day in control DS-treated and DS-treated stressed groups. From day 1 to day 14, mice were subjected to nightly rat exposure stress. Between the hours of 12:00 and 18:00, they were exposed to social defeat for 30 min and restraint stress for 2 h, with an inter-session interval of at least 4 h. After the last stress session, mice were re-tested in the sucrose preference test, and stressed groups were classified as resilient or anhedonic based on a 65% criterion of anhedonia. After that, a tail suspension test was carried out, and animals were studied for a coat disintegration score. On day 15, a nest building assessment and a splash test were performed, followed by the marble test on day 16 and the Y-maze test on days 17–19. On day 20, after the last behavioural assessment, mice were sacrificed, and the brains were dissected. The prefrontal cortex (PFC), hippocampus, raphe, striatum, and motor cortex were harvested to be used for a subsequent RNA isolation and RT-PCR assay, and hippocampi were used in the Illumina assay (qRT-PCR—quantitative reverse transcription polymerase chain reaction).

Here, C57BL/6 mice were first studied in a sucrose preference test, and then they underwent rat exposure, restraint, and social defeat for 2 weeks (Figure 1). A cohort of mice naïve to stress and CMS mice received DS at 50 mg/kg/day via drinking water alongside exposure to a stress regimen. A previous study demonstrated the efficacy of 25 mg/kg/day of DS administrated intraperitoneally starting 7 days prior to the stress [28]. The dosing scheme in this study was selected to use more clinically relevant conditions of drug administration—that is, with an onset at the start of the stress challenge and an application of DS per os. By the end of the stress procedure, the animals were re-tested for sucrose preference and, according to a previously validated 65% criterion of sucrose preference [47,71,73], they were assigned to the susceptible or resilient-to-anhedonia groups. The mice were studied for immobility behaviour in the tail suspension test, grooming behaviour in a splash test, nest building, and coat state, as described elsewhere [57,74]. Their spatial learning was investigated in the Y-maze, as described previously [75]. The hippocampus-dependent performance was further studied in the marble T-test [69]. Following the termination of behavioural experiments, the mice were sacrificed, and their hippocampus, prefrontal cortex (PFC), raphe dorsalis, striatum, and motor cortex (MC) were dissected for subsequent molecular studies. These regions were collected to address potential region-specific differences that might be associated with stress response and the effects of DS.

Using real-time quantitative reverse transcription polymerase chain reaction (RT-PCR), we studied mRNA regional concentrations of pro-inflammatory molecules, interleukin-1β (*Il-1β*), tumour necrosis factor (*Tnf*), and cyclooxygenase-1 (*Cox-1*), for which expression is well documented to be related to altered mitochondrial functions during stress and the development of MDD-like syndrome; they were also implicated in the CMS mouse model [57,76,77,78]. Additionally, we investigated gene expression of the serotonin transporter (5-*htt*) and the 5-*ht2a* serotonin receptor (5-*ht2a-r*), as changes in these genes are known to accompany MDD in clinic and in depressive-like syndrome in rodent models of stress [79]. There were profound changes among the studied brain structures in the hippocampus, in which we also found a decrease in 5-*ht2a-r*, a key MDD marker based on anatomical and pharmacological studies [79]. Given these differences and the fact that the significant behavioural alterations caused by stress and dosing with DS were related to hippocampus-dependent performance, we then studied the expression of plasticity factors and the mitochondrial complexes pathways specifically in this brain area.

Therefore, we selected well-established markers of hippocampal plasticity for the RT-PCR assay: calmodulin-kinase 4 (*Camk4*), calmodulin-kinase 2 (*Camk2*), protein kinase A (*Pka*), adenylyl cyclase type 1 (*Adcy1*), adenylyl cyclase type 2 (*Adcy2*), cyclic AMP response element-binding protein A receptor (*creb-ar*), cyclic AMP response element-binding protein substrate (*Creb-s*), N-methyl-D-aspartate receptor subunit 2R (*Nmda-2r-ar*), and N-methyl-D-aspartate receptor subunit 2R- Substrate (Nmda-2r-s) [80,81,82,83,84,85,86]. Because previous extensive experiments in non-challenged DS-treated mice have shown a lack of any effects on brain expression of *Nmda* receptor subunits [46,47,48], inflammation-related markers, markers of mitochondrial functions [48], and the neurotrophic/plasticity molecule *Igf2* [46], we omitted a non-stressed, DS-treated group from this study. Finally, we performed Illumina sequencing on hippocampal RNA and found significant gene expression changes. We analysed the data to compare mitochondrial function between the anhedonic and resilient subgroups of mice and also among the DS-treated mice.

## 2. Materials and Methods

### 2.1. Animals

Our experiments were performed using 3-month-old male C57BL/6J mice. Three-month-old male CD1 mice were used as intruders for social defeat stress and 2.5-month-old Wistar rats were used for predator stress. All animals were obtained from the certified provider of Charles River (Stolbovaja, RAS, Moscow region (http://www.spf-animals.ru/, accessed on 12 July 2023). C57BL/6J mice were housed individually for 10–14 days before the start of the experiments; CD1 male 3-month-old mice were housed five per cage during this study; rats were housed in groups of five before the experiment and then individually. Animals were kept under a 12 h light–dark cycle (lights on: 20:00 h) with food and water ad libitum using controllable laboratory conditions (22 ± 1 °C, 55% humidity). All experiments were carried out in accordance with the European Communities Council Directive for the care and use of laboratory animals 2010/63/EU upon approval by the Ethical Committee of MSMU #11-18-2018/2019 on animal care and welfare and in compliance with ARRIVE guidelines (http://www.nc3rs.org.uk/arrive-guidelines, accessed on 12 July 2023).

### 2.2. Study Flow

This experiment used a previously established 2-week stress protocol that was adapted from a described method [57,71]. The stress regimen comprised nighttime rat exposure (between the hours of 20:00 and 09:00) combined with the daytime application of two stressors—social defeat and restraint stress, a selection of which was applied in a semi-random manner [57]. Specifically, between the hours of 12:00 and 18:00, social defeat was applied for 30 min, and restraint stress was applied for 2 h, with an inter-session interval of at least 4 h.

With the DS chronic dosing, this compound (Buddha Biopharm, Kuopio, Finland) was administrated concomitantly with the stress protocol per os via drinking water at a dose of 50 mg/kg/day, as described elsewhere [46,48]. Among the drug-free animals, 9 naive control mice were used, and the mice were subjected to stress. Among the DS-dosed mice, 8 were non-stressed and 16 were assigned to a stress group. At the baseline, control and stress groups of mice were balanced upon their sucrose preference (see below). The sucrose preference test was repeated during the 2nd week (day 14) of stress exposure after the termination of the stress procedure. Mice were assigned to resilient or anhedonic cohorts according to their sucrose preference: whether it had not decreased or had decreased lower than the minimum value in a control group (65%). Thereafter, all mice were studied in relation to the the tail suspension test (day 14), coat disintegration score (day 14), nest building (day 15), and self-grooming in the splash test (day 15). Their cognitive abilities were then investigated in the marble test (day 16) and the spatial version of the Y–maze (day 17–19).

All mice were sacrificed 14 h after the termination of the last behavioural session, and their PFC, hippocampus, raphe striatum, striatum, and motor cortex were dissected to be used for a subsequent PCR study (Figure 1). Hippocampi were used for the Illumina assay.

### 2.3. Chronic Stress Procedure and Determination of Anhedonia

In this study, mice were subjected to three different stressors (rat exposure, restraint stress, and social defeat) over 2 weeks, as described elsewhere [77,78]. For rat exposure stress, mice were introduced to a transparent glass cylinder (15 cm high × Ø 8 cm) and placed into the rat cage (15 h exposures were performed between 18:00 and 09:00), as described previously [87,88]. During restraint stress, mice were placed into a small container (50 mL Falcon Tube) with space for breathing but no space for free movement. After 2 h, the animals were removed and returned to their home cage.

Social defeat procedures took place during the dark phase; to enable visual control over the resident–intruder confrontation, the test was carried out under red light. In a preliminary test, aggressive individuals of the CD1 mouse strain that were able to attack the counter-partners in less than 60 s without injuring them were selected for this procedure; these animals were introduced in the home cages of mice from the stress group during social defeat sessions for 5 min. During social defeat stress, test mice typically showed flight response, submissive posture, and vocalisation. Pairs of animals were carefully observed in order to exclude any physical harm. In rare cases of its incidence, aggressive individuals were immediately removed from the cage of resident mice. After a 5 min period of social defeat, C57BL/6 mice were introduced into small containers and again placed inside the CD1 cage, where they stayed for a 3 h period. Thereafter, a 5 min social defeat procedure was repeated again. In order to randomise the procedure, the same pairs of C57Bl6 and CD1 mice were never put together.

### 2.4. Sucrose Preference Test

Mice were given eight hours of free choice between two bottles of 1% sucrose and standard drinking water. At the beginning and end of the period, the bottles were weighed and consumption was calculated. The beginning of the test started with the onset of the dark (active) phase of the animals’ cycle (i.e., at 9:00). To prevent the possible effects of side preference in drinking behaviour, the position of the bottles in the cage was switched at 4 h (halfway through testing). No previous food or water deprivation was applied before the test. To minimise the spillage of liquids during the sucrose test, bottles were filled in advance and kept in the upside down position for at least 12 h prior to testing. In order to balance the air temperature between the room and the drinking bottles, they were kept in the same room where the testing took place. This measure prevents the physical effect of liquid leakage resulting from growing air temperature and pressure inside the bottles when they are filled with liquids that are cooler than the room air. In order to decrease variability in sucrose consumption during the very first sucrose test (baseline measurement), a day before, animals were allowed to drink 2.5% sucrose solution in a one-bottle paradigm for 2 h.

The percentage preference for sucrose was calculated using the following formula:

$$Sucrose\;Preference=100\times \frac{Volume(Sucrose\;solution)}{Volume\left(Sucrose\;solution\right)+Volume(Water)}$$

No mice from control groups ever exhibited a preference for sucrose of <65% and, accordingly, mice exhibiting a sucrose preference of <65% were defined as susceptible. Mice that had undergone stress but maintained a sucrose preference of >65% were defined as resilient. Other conditions of the test were applied as described elsewhere [57].

### 2.5. Tail Suspension Test

Mice were subjected to the tail suspension test by being hung by their tails with adhesive tape to a rod 50 cm above the floor for 6 min. Animals were tested in a dark room where only the area of the modified tail suspension construction was illuminated by a spotlight from the ceiling; the lighting intensity on the height of the mouse position was 5 Lux. The total duration of immobility was scored according to the protocol that was previously validated with Noldus EthoVision XT 8.5 (Noldus Information Technology, Wageningen, Netherlands; Ref. [74]). In accordance with the commonly accepted criteria of immobility, the immobility behaviour was defined as the absence of any movements of the animals’ heads and bodies.

### 2.6. Evaluation of Coat Disintegration

This assay was carried out as described elsewhere [88]. All mice were scored for their coat state prior to stress and 15 h after the termination of the last stressor (5—excellent condition, 1—poor condition).

### 2.7. Splash Test

This test was performed as described elsewhere [74,89]. A 10% sucrose solution was spread on the dorsal surface of the mouse coat, which, because of its high viscosity, induces lasting grooming behaviour in mice. The parameters generally accepted for splash tests, i.e., latency of the first episode of grooming, number of grooming episodes, and duration of grooming behaviour, were scored.

### 2.8. Evaluation of Nest Building Performance

In order to carry out this test, a piece of facial tissue was placed in each cage at 18.00, and the mouse’s performance to build up a nest was evaluated the next morning at 9:00. This activity was scored as follows: 5—excellent performance, 1—poor performance.

### 2.9. Marble T-Test

All experimental groups were tested for pellet displacement in a marble test as described elsewhere [90,91,92]. A tendency to displace small objects, e.g., small stones or food pellets, from a tube inside the cage is species-specific in mice and has been demonstrated to depend on an intact hippocampal formation. Using a paper tube (internal diameter 4 cm, length 10 cm) filled with 20 food pellets and placed in the middle of a home cage (21 cm × 27 cm × 14 cm), the number of food pellet displaced by each mouse was assessed every 15 min during 1 h.

### 2.10. Y-Maze

The Y-maze construction was an apparatus made from black Plexiglas, which consisted of three arms (40 × 6 × 10 cm) with an angle of 120° between each symmetrical arm (Technosmart, Rome, Italy). The illumination strength was 5 lx. To allow spatial orientation of the mice, paper figures of different shapes (approx. size 20 × 40 and 20 × 30 cm) were placed on the walls of the laboratory. Validation experiments have demonstrated that the learning of the task by C57BL6 mice occurs starting from day 3 of acquisition in which two trials per day are performed [75]. At the ends of the arms, two bottles, one filled with water and another empty, were placed in a position that was adjusted to allow drinking. Before the first session, mice were water deprived for 18 h. Following a previously established protocol, two 10 min training sessions per day spaced 1 h apart were carried out for 3 consecutive days. Therefore, a mouse was placed at the starting point of the apparatus and allowed to explore either arm of the maze containing the bottles. About one half of each experimental group of animals was trained to receive a water reward from either the left-hand or right-hand bottle. Each mouse was allowed to drink for up to 10 min in each training session. When no drinking behaviour was observed by the end of the training day, mice were allowed to drink after the termination of behavioural testing while in the Y-maze. The body weight of all mice was monitored throughout the testing period. Previous studies using this Y-maze protocol showed a lack of negative effects of the drinking schedule on body weight [75]. Based on a study with memory enhancers, we used previously validated parameters of learning in this task: the latency to reach the filled bottle and the percentage of correct choices for the arm containing this bottle [75].

### 2.11. Administration of DS

Dicholine Succinate (Biddha Biopharm, Helsinki, Finland) was dissolved in tap water. Mice from control non-stressed and stressed groups were housed with DS solution during the entire two-week period of the stress protocol. The dose and concentration of DS in drinking water were based on previous studies [46,48,87]. We measured liquid intake while dosing the animals via drinking water, which was carried out daily during the first three days of the experiment and followed by weekly measurements, as described elsewhere [77,78].

### 2.12. Culling and Brain Dissection

Mice were terminally anaesthetised with isoflurane inhalation and sacrificed through cervical dislocation for subsequent material collection. For the gene expression assay, mice were perfused with ice-cold saline via the left ventricle, brains were removed, and the hippocampi, PFC, raphe dorsalis, striatum, and motor cortex were dissected as described elsewhere [77,78] and stored at −80 °C until use, as described elsewhere.

### 2.13. RNA Extraction and RT-PCR

First strand cDNA synthesis was performed using random primers and Superscript III transcriptase (Invitrogen, Darmstadt, Germany). First, 1 μg of total RNA was converted into cDNA. Quantitative PCR for selected genes and the housekeeping gene glyceraldehyde 3-phosphate dehydrogenase (*Gapdh*) was performed using the SYBR Green master mix (Bio-Rad Laboratories, Philadelphia, PA, USA) and the CFX96 Real-time System (Bio-Rad Laboratories, Philadelphia, PA, USA). Sequences of primers were used as previously described in our earlier studies [57,77,78,93]. Data were normalised to *Gapdh* mRNA expression and calculated as relative-fold changes compared to control mice, as described elsewhere [57]. Specifically, the results of RT-PCR measurement were expressed as Ct values, where Ct is defined as the threshold cycle of the PCR at which the amplified product was 0.05% of the normalised maximal signal. We used the comparative Ct method and computed the difference between the expression of the gene of interest and GAPDH in each cDNA sample (2^−ΔΔCt^ method). Data are given as expression-folds compared to the mean expression values in control mice.

### 2.14. Illumina Gene Expression Profiling and Ingenuity Pathway Analysis

Gene expression profiling was performed using Illumina technology (IntegraGen, Evry, France) and the Ingenuity Pathway Analysis program (Ingenuity Systems, Redwood city, CA, USA) utilising the hippocampi of control versus stressed, anhedonic, and non-anhedonic mice, as described elsewhere [72,87]. The samples were assigned to the chips in a random order, with the constraint that no two samples from the same group were used for the same chip to avoid confounding the experimental groups with the chips. Obtained microarray data were analysed using standard analysis procedures, which included assessment of the overall quality of array data and statistical evaluation of differentially expressed genes. Next, the quality of array data was confirmed, and the Gene Chip Operating System was used to calculate signal intensities, detection calls, and their associated *p* values for each transcription array. Gene expression was normalised to the expression of the housekeeping gene, *Gapdh*, due to its stable expression, and calculated as fold changes of the control group of non-stressed mice. Differences in gene expression between groups were evaluated using two-way ANOVA. Illumina data were imported into Partek Genomics Suite and quantile normalised. Arrays that appeared as outliers on PCA were removed from the dataset. Comparisons between experimental groups were carried out in Partek Genomics Suite, and ANOVA with appropriate contrasts was used. *p*-values were adjusted for multiple testing using the step-up False Discovery Rate (FDR). The following criteria were used to select differentially expressed genes at different stringency levels: Strict: F DR < 0.05 and |fold change| > 2; Medium: FDR < 0.1 and |fold change| > 1.5; Loose: unadjusted *p*-value < 0.001 and |fold change| > 1.3; Very loose: unadjusted *p*-values < 0.01 and no fold change threshold (only used when more stringent selection criteria yielded zero or very few hits). In the current analysis, ‘medium‘ criteria were applied. Each group comprised 5 animals in this study. Next, gene expression changes that corresponded based on the medium stringency criteria applied here were analysed using Mitochondrial Oxidative Phosphorylation Gene Pathway Enrichment analysis and the Ingenuity Pathway Analysis program, as described elsewhere [72]. This program organises large groups of genes into coherent networks where proteins interact both physically and functionally based on validated information in many biological systems. These networks bring together several biological functions, including oxidative phosphorylation in mitochondria that were analysed in our study.

### 2.15. Statistical Analysis

The data were analysed using a statistical software package (GraphPad PRISM 9.1.0, San Diego, CA, USA). For normality testing, the Shapiro–Wilk test was used. For normally distributed data, one-way and two-way ANOVA were applied where appropriate. One-way ANOVA was followed by Tukey’s test or Dunnett’s T3 test where appropriate. The two-way ANOVA test was followed by the post hoc Tukey’s test for interaction testing and Šídák’s test for the main effect only. Three-way repeated measures ANOVA with post hoc Tukey’s test were performed for the Y-maze. Geisser–Greenhouse correction was applied for repeated measures ANOVA. The Kruskal–Wallis test with Dunn’s post hoc test were used for data lacking normal distribution. Qualitative data were analysed using the two-tailed Fisher’s exact test. The level of confidence was set to 95% (*p* < 0.05). Data are shown as boxplots with median, first, and third quartiles and minimum to maximum whiskers.

## 3. Results

### 3.1. Sucrose Preference Test

**Figure 2:**
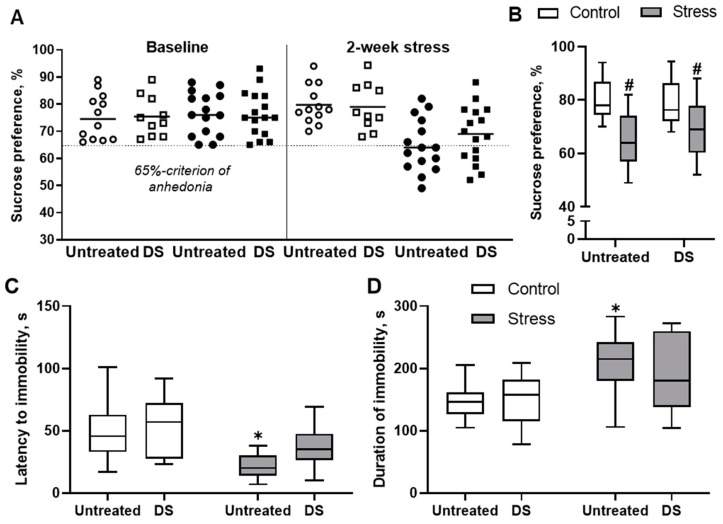
Hedonic-like behaviour and helplessness in untreated and DS-treated stressed mice. In the sucrose preference test, (**A**) mice had no group differences in sucrose preference before the stress procedure and (**B**) significantly lower sucrose preference in both stressed groups than in treatment-matched non-stressed mice. In the tail suspension test (**C**), significantly increased duration of immobility and (**D**) significantly lowered latency to the first immobility episode were found in the untreated, stressed group compared to the untreated control animals. Open symbols—non-stressed groups, closed symbols—stress groups. * *p* < 0.05 compared to untreated control group, # *p* < 0.05 compared to respective untreated or DS-treated controls; two-way ANOVA with post hoc Tukey’s test and Šídák’s test. Data are presented as boxplots with median, first, and third quartiles and minimum to maximum whiskers. Untreated control *n* = 12, DS-treated control *n* = 10, untreated stressed group *n* = 15, DS-treated stressed group *n* = 16.

In the sucrose preference test, a well-established behavioural assay for sensitivity to reward [57,69], two-way ANOVA, does not reveal any significant differences in sucrose preference measured before the start of the stress procedure (all *p* > 0.05, two-way ANOVA, Figure 2A). In turn, after two weeks of the stress procedure, there is a significant effect of stress found (F1,49 = 23.25, *p* < 0.01, two-way ANOVA), but not of the treatment factor (F1,49 = 0.40, *p* = 0.53) or the factors’ interaction (F1,49 = 0.87, *p* = 0.36). Post hoc analysis shows that in both stressed groups, sucrose preference is significantly decreased compared to treatment-matched control groups (untreated: *p* < 0.01; DS-treated: *p* = 0.02, Šídák’s test, Figure 2B). Based on the results of this test, mice have been stratified to anhedonic (sucrose preference less than 65%) and resilient groups. A 65% criterion is based on the fact that control, unstressed animals do not display values of sucrose preference below this figure. The number of mice classified as anhedonic is non-significantly lower among the DS-treated stressed group compared with the number of anhedonic animals among untreated, stressed mice (*p* > 0.05, Fisher’s exact test, Supplementary File, Figure S1). Importantly, there are no group differences in intake of liquids between mice housed with plain water and DS across this study (*p* > 0.05), which is consistent with our previous results [47,48] and suggests that drinking behaviour in the sucrose test is not compromised. Hence, stress exposure significantly affected sensitivity to a reward, which is a sign of an MDD-like state, in both untreated and DS-treated groups of mice.

### 3.2. Tail Suspension Test

This test was applied to study behavioural signs of helplessness, a feature of clinical MDD, in experimental groups of mice [69]. Regarding the duration of immobility and the latency to immobility in the tail suspension test, two-way ANOVA reveals a significant main effect of stress (F1,49 = 15.1, *p* < 0.01 and F1,48 = 17.8, *p* < 0.01, respectively, two-way ANOVA, Figure 2C,D) but not of the DS treatment or interaction (F1,49 = 0.37, *p* = 0.55 and F1,49 = 0.70, *p* = 0.41). Post hoc analysis shows a significantly increased time of immobility and significantly decreased latency to immobility in untreated, stressed mice compared to untreated control mice (both *p* < 0.01, Šídák’s test). Thus, stress causes signs of depressive-like behaviour, such as helplessness, which are partly reversed by DS treatment. We have found no group differences between untreated and DS-treated subgroups with respect to the assignment of stressed mice to anhedonic or non-anhedonic cohorts. In other words, there were no significant differences in the parameter of immobility between untreated anhedonic and DS-treated, anhedonic groups or between untreated resilient and DS-treated resilient groups, respectively (see Supplementary File, Figure S2A,B).

### 3.3. Marble T-Test

**Figure 3:**
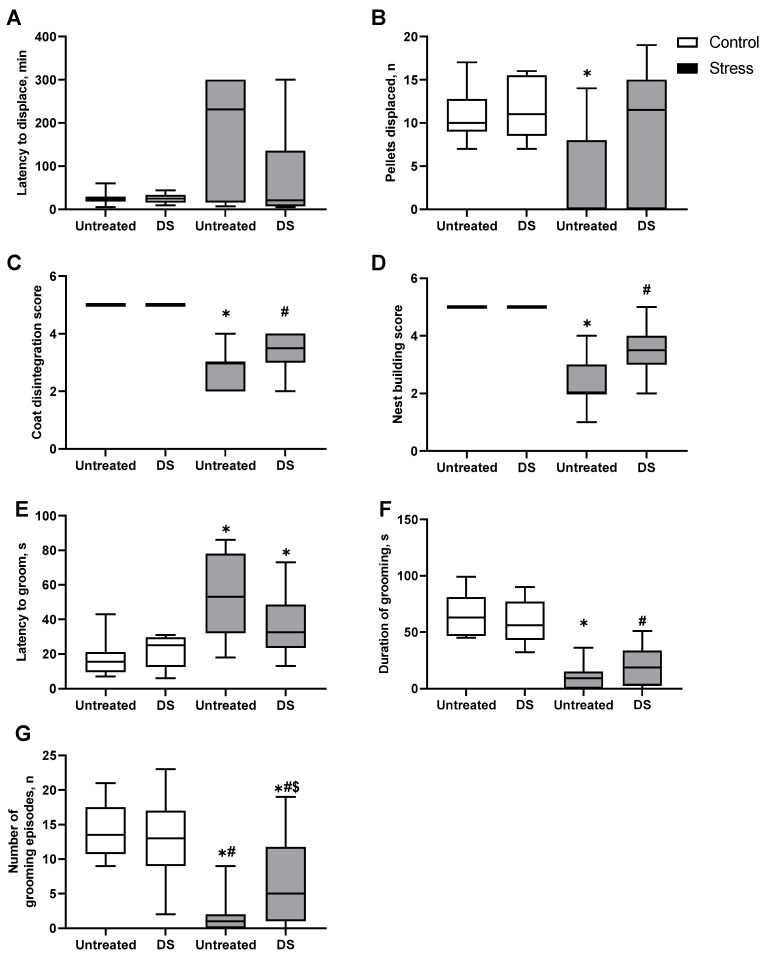
Depressive-like and hippocampus-dependent behaviours in untreated and DS-treated mice. In the pellet displacement test, (**A**) no significant changes were found in the latency to displace the first pellet, although there was (**B**) a significant decrease in the number of pellets that were displaced by untreated, stressed mice in the first 20 min of the test compared to the untreated controls. In both stressed groups, there were significant decreases in (**C**) nest building scores and (**D**) coat scores in comparison with treatment-matched control groups. In the splash test, stressed animals showed (**E**) significantly increased latency to the first grooming episode and (**F**) significantly decreased total duration of grooming, whereas (**G**) the numbers of grooming episodes in both stressed groups were significantly lower than in either of the control groups; in the untreated, stressed mice, this measure was also significantly decreased compared to the DS-treated stressed animals. * *p* < 0.05 compared to the untreated control group, # *p* < 0.05 compared to the DS-treated control group, $ *p* < 0.05 compared to the untreated, stressed group. Sucrose splash test: two-way ANOVA with post hoc Tukey’s test and Šídák’s test. Pellet displacement test, nest building, and coat scores: Kruskal–Wallis test with post hoc Dunn’s test. Please note that in the control, non-stressed groups, the coat disintegration score and nest building score were unaltered, and all were equal to a maximum value. Data are presented as boxplots with median, first, and third quartiles and minimum to maximum whiskers. Untreated control *n* = 12, DS-treated control *n* = 10, untreated, stressed group *n* = 15, DS-treated, stressed group *n* = 16.

To study how hippocampus-dependent performance was potentially altered by stress and DS treatment, we employed the marble T-test (pellet displacement test), which is a well-established model in mice [69]. In this experiment, the latency to displace the first pellet is not significantly different between groups (*p* = 0.11, Kruskal–Wallis test, Figure 3A). However, the number of pellets displaced during the first 20 min of the test is significantly different between groups (*p* < 0.01, Kruskal–Wallis test): untreated, stressed mice displaced significantly fewer pellets than untreated control mice (*p* = 0.03, Dunn’s test Figure 3B). No significant differences are found in the stressed DS-treated group compared to the DS-treated control group (*p* > 0.99, Dunn’s test) or the untreated control animals (*p* > 0.05, Dunn’s test). The latency to displace the first pellet is significantly elevated in the untreated, anhedonic group but not in the DS-treated, anhedonic mice; the number of displaced pellets is diminished in both untreated and DS-treated, anhedonic mice in comparison to the untreated and DS-treated, resilient groups, respectively (see Supplementary File, Figure S2F). Such differences indicate possible impairments of hippocampus-dependent behaviours, which are at least partially ameliorated by the DS treatment.

### 3.4. Coat Disintegration and Nest Building Scores

The physical state and self-care of stressed mice were investigated by scoring their coat disintegration and nest building; previous studies have shown a decrease in these behaviours to accompany the anhedonic state in mice [47,69]. Both the coat score and nest building score are significantly altered (both *p* < 0.01, Kruskal–Wallis test). Both scores are significantly decreased in stressed groups compared to corresponding non-stressed groups (all *p* < 0.01, Dunn’s test, Figure 3C,D). In these measures, there are no significant differences found between untreated and DS-treated control groups, nor between untreated and DS-treated stressed groups (all *p* > 0.05, Dunn’s test). However, the coat score is significantly lowered in untreated, anhedonic mice but not in the DS-treated, anhedonic animals (see Supplementary File, Figure S2H). In nest building, no subgroup differences are found using post hoc Dunn’s test between untreated and DS-treated mice with respect to the development of anhedonia, i.e., between untreated, anhedonic and DS-treated, anhedonic groups, or between untreated, resilient and DS-treated, resilient groups, respectively (see Supplementary File, Figure S2H,I).

### 3.5. Splash Test

The self-care behaviour of stressed mice was additionally investigated in the splash test because a suppression of grooming behaviour in this paradigm was previously found to correlate with signs of anhedonia and helplessness in a mouse depression model [74]. The latency to grooming is significantly affected by the interaction between stress and DS treatment factors (F1,48 = 27.3, *p* < 0.01, two-way ANOVA). In both stressed groups, latency to grooming is longer in stressed animals than in untreated control mice (stressed untreated: *p* < 0.01, stressed DS-treated: *p* = 0.03, Šídák’s test; Figure 3E). The duration of grooming episodes is significantly affected by the stress factor (F1,48 = 106.5, *p* < 0.01, two-way ANOVA) but not by the DS treatment (F1,48 = 0.13, *p* = 0.72) or interaction (F1,48 = 2.48, *p* = 0.12). In both stressed groups, this parameter is significantly lower compared to the corresponding control groups (both *p* < 0.01, Šídák’s test Figure 3F). The number of grooming episodes is affected by the interaction between stress and treatment factors (F1,48 = 44.77, *p* < 0.01, two-way ANOVA). Post hoc analysis reveals a significant decrease in the number of grooming episodes in both stressed groups compared to the untreated control group (both *p* < 0.01, Tukey’s test) and compared to the DS-treated control mice (stressed, untreated: *p* < 0.01, stressed DS-treated: *p* = 0.02, Tukey’s test, Figure 3G). The number of grooming episodes in untreated, stressed mice is also significantly decreased compared to DS-treated stressed mice (*p* = 0.04, Tukey’s test). A comparison of untreated and DS-treated cohorts of mice classified based on a criterion of anhedonia shows a higher number of grooming episodes in DS-treated, resilient mice compared with non-treated, resilient mice (see Supplementary File, Figure S2E); no other differences with regard to a comparison of anhedonic vs. resilient subgroups are found. These data suggest that stress suppresses a self-care behaviour in the experimental groups of mice and that the administration of DS has partially ameliorated this measure.

### 3.6. Y-Maze Learning

**Figure 4:**
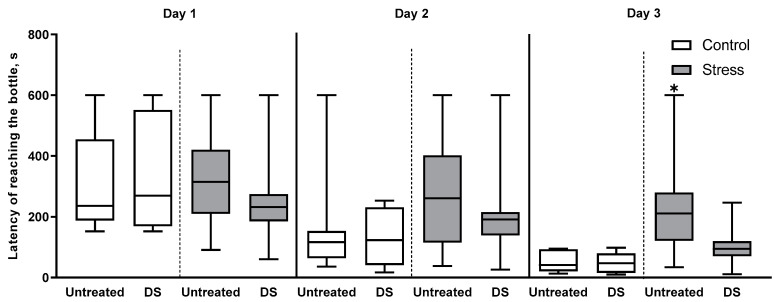
Decreased performance in Y-maze in untreated, stressed mice on day 3. Latency to reach the bottle on day 3 was significantly increased in untreated, stressed mice compared to the untreated control group. * *p* < 0.05 compared to the untreated control group. Three-way repeated measures ANOVA with post hoc Tukey’s test. Data are presented as boxplots with median, first, and third quartiles and minimum to maximum whiskers. Untreated control *n* = 8, DS-treated control *n* = 8, untreated, stressed group *n* = 15, DS-treated, stressed group *n* = 16.

To study potential deficits in hippocampus-dependent performance further, we employed a paradigm of spatial learning in the Y-maze, in which mice were trained for three consecutive days to locate a correct arm containing a drinking bottle [75]. Three-way repeated measures ANOVA shows a significant effect of interaction between day and stress factors (F2,86 = 4.1, *p* = 0.02, three-way repeated measures ANOVA, Figure 4). The effects of treatment factor, day × treatment interaction, treatment × stress interaction, and day × treatment × stress interaction do not reach significance (all *p* > 0.05, three-way repeated measurements ANOVA). A post hoc test reveals significantly longer latency of reaching the bottle on day three in the untreated, stressed group compared to the untreated control mice (*p* = 0.01, Tukey’s test). Such a difference on day three is absent between DS-treated stressed and control groups (*p* = 0.22, Tukey’s test). Other post hoc comparisons also do not reveal any significant differences between groups differing by only one factor (all *p* > 0.05, Tukey’s test). In this test, there are no group differences between untreated and DS-treated subgroups in relation to the assignment of stressed animals to anhedonic or resilient cohorts, i.e., between untreated, anhedonic and DS-treated, anhedonic groups, or between untreated, resilient and DS-treated, resilient groups, respectively (*p* > 0.05). Together, a lack of significant differences in the performance of DS-treated control and stress groups suggests that the applied pharmacological treatment rescued stress-induced impairment in the spatial memory of mice.

### 3.7. The mRNA Expression of the Inflammation Markers and Serotonergic System Proteins in Selected Brain Regions

**Figure 5:**
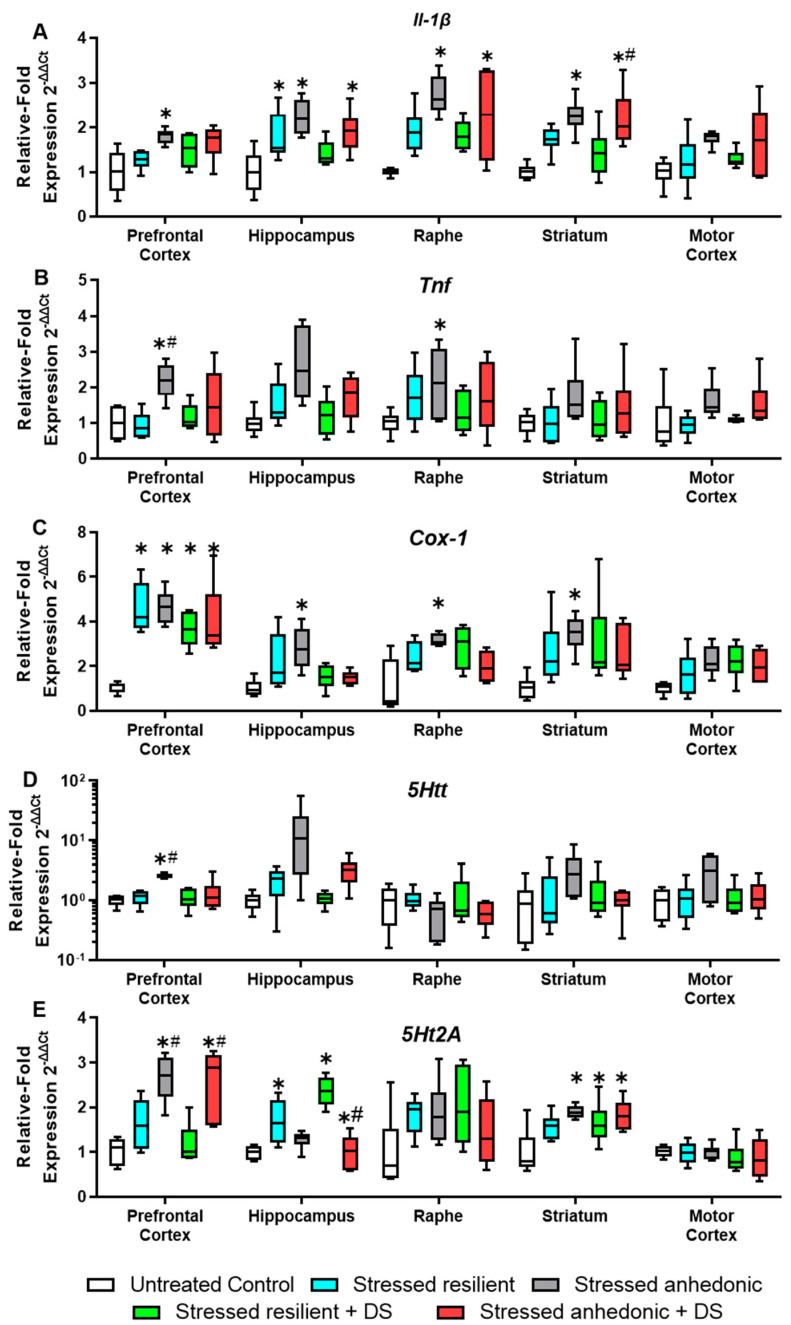
Inflammatory markers and 5-HT-related genes expression changes across various brain regions of stressed untreated and DS-treated mice. (**A**) Significant increases in the *Il-1β* expression were observed in the PFC, striatum, hippocampus, and raphe of most stressed groups; however, in the PFC, DS treatment prevented such changes in both resilient and anhedonic, stressed groups and in the striatum, hippocampus, and raphe in the resilient, stressed group. (**B**) The *Tnf* expression was significantly increased in the PFC and raphe only in the untreated, stressed, anhedonic group. (**C**) *Cox-1* expression was significantly increased in all of the stressed groups in the PFC independently of treatment or anhedonic status. In the striatum, hippocampus, and raphe, such changes were observed in the untreated, anhedonic animals only. (**D**) *5-Htt* expression was significantly increased only in the PFC of the untreated, anhedonic mice. (**E**) In the PFC, striatum, and hippocampus, massive changes were observed in the expression of the 5-*Ht2A* receptor. It was significantly increased in the PFC of both anhedonic groups, in the striatum of all groups, except untreated, resilient mice, and in the hippocampus, with the exception of stressed, anhedonic mice. * *p* < 0.05 compared to control group, # *p* < 0.05 compared to treatment-matched resilient group. Data are presented as boxplots with median, first, and third quartiles and minimum to maximum whiskers. One-way ANOVA with Tukey’s test and Dunnett’s T3 test. All groups *n* = 6.

Because previous studies on mice have demonstrated an over-production of pro-inflammatory markers associated with exposure to stress and susceptibility to an anhedonic-like state [42,48,66,75,79,81], we addressed this issue in our current work, as well. In the *Il-1β* expression, one-way ANOVA reveals significant differences in the PFC, MC, striatum, hippocampus, and raphe (F4,25 = 5.1, *p* < 0.01; W4,11.96 = 8.3, *p* < 0.01; F4,25 = 8.7, *p* < 0.01; F4,25 = 7.0, *p* < 0.01; W4,10.57 = 29.8, *p* < 0.01, respectively, one-way ANOVA, Figure 5A; all statistical data for group comparisons for this and other genes in this section are presented in Supplementary Table S2). In the PFC, the expression of *Il-1β* is significantly increased in both the untreated (*p* < 0.01, Tukey’s test) and the DS-treated (*p* = 0.02), stressed, anhedonic group compared to the control group. In the MC, a significant increase in *Il-1β* expression compared to the control group is found in the untreated, stressed, anhedonic group (*p* < 0.01, Dunnett’s T3 test). In the striatum, a significant increase in expression compared to the control group is found in untreated (*p* < 0.01, Tukey’s test) and DS-treated, stressed, anhedonic groups (both *p* < 0.01, Tukey’s test), and the expression is also significantly increased in the DS-treated, stressed, anhedonic group compared to the DS-treated, stress-resilient group (*p* = 0.04). The expression of the *Il-1β* in the hippocampus is significantly increased in the stressed resilient, stressed anhedonic, and stressed anhedonic DS-treated groups compared to the control (*p* = 0.04, *p* < 0.01, *p* = 0.01, respectively, Tukey’s test). Notably, no significant difference is found between the control group and the stressed, resilient, DS-treated group (*p* = 0.50). In the raphe, the expression of *Il-1β* compared to the control group is significantly increased in the stressed resilient, stressed anhedonic, and stressed, resilient, DS-treated groups (*p* = 0.04, *p* < 0.01, *p* = 0.01, respectively, Dunnett’s T3 test).

The *Tnf* expression is significantly altered in the PFC and hippocampus (F4,25 = 5.0, *p* < 0.01; F4,25 = 5.6, *p* < 0.01, respectively, one-way ANOVA, Figure 5B). In the PFC, the stressed anhedonic group has a significantly increased TNF expression compared to the control group (*p* = 0.1, Tukey’s test) and the stressed, resilient group (*p* < 0.01). In the hippocampus, expression in the stressed anhedonic group is significantly increased in comparison to the control group.

The expression of the *C_OX_-1* is significantly altered in all of the structures: PFC (F_4,25_ = 14.1, *p* < 0.01, one-way ANOVA, Figure 5C), MC (F_4,25_ = 2.9, *p* = 0.04), striatum (F4,25 = 3.0, *p* = 0.03), hippocampus (F4,25 = 5.1, *p* < 0.01), and raphe (F4,25 = 3.8, *p* = 0.02). Significant increase in the *Cox-1* expression in the PFC is revealed in all stressed groups compared to the control mice (all *p* < 0.01, Tukey’s test). In the striatum, hippocampus, and raphe, expression is significantly higher in the stressed anhedonic group compared to the control (*p* = 0.02, *p* < 0.01, and *p* = 0.02, respectively). In addition, in the hippocampus, a significantly decreased expression of the *Cox-1* is found in the stressed, anhedonic, DS-treated group compared to the untreated, stressed, anhedonic mice (*p* < 0.05). Notably, brain expression of inflammatory factors was overall lower in the DS-treated stressed groups. In resilient mice, hippocampal expression of *Cox-1* was significantly elevated in the untreated cohort of mice, but not in DS-treated animals. A comparison of anhedonic subgroups of mice showed elevated gene expression of *Il-1β* and *Tnf* in the PFC and of *Cox-1* in the hippocampus and striatum of untreated animals that was not revealed in anhedonic, DS-treated mice. These findings suggest anti-inflammatory effects of the administration of DS.

Because the changes in 5-HT-mediated neurotransmission and, specifically, in the functions of the 5-HT2A receptor are particularly well-established characteristics of molecular changes in the brains of MDD patients that are recapitulated in mouse models of depression [70,72,73,77,78], here, we studied the gene expression of *5-Htt* and 5-*Ht2A* receptors. Significant differences in the *5-htt* expression are found in the PFC, MC, and hippocampus (F4,25 = 11.7, *p* < 0.01; F4,10.23 = 3.7, *p* = 0.04; W4,11.56 = 4.1, *p* = 0.03, respectively, ordinary one-way ANOVA, Figure 5D). Significant group differences in the expression of the *5-Htt* are revealed only in the PFC, where the expression in the stressed, anhedonic group is significantly increased in comparison to all other groups (all *p* < 0.01, Tukey’s test). In the MC and the hippocampus, there are no significant group differences (all *p* > 0.05).

One-way ANOVA reveals significantly altered expression of the *5*-*Ht2A* receptor in the PFC, striatum and hippocampus (F4,25 = 12.3, *p* < 0.01; F4,25 = 5.6, *p* < 0.01; F4,25 = 18.6, *p* < 0.01, respectively, one-way ANOVA, Figure 5E). Compared to the control group, the expression of 5-*Ht2A* in the PFC is significantly increased in the stressed anhedonic group and stressed, anhedonic, DS-treated animals (both *p* < 0.01, Tukey’s test). The expression is also significantly increased in the stressed, anhedonic animals compared to the stressed, resilient group (*p* = 0.02) and in the stressed, anhedonic, DS-treated mice compared to stressed, resilient, DS-treated animals (*p* < 0.01). In the striatum, a significant increase in the *5*-*Ht2A* expression is observed in all groups except the stressed, resilient group (stressed, anhedonic group *p* = 0.04; stressed, resilient, DS-treated group and stressed, anhedonic, DS-treated group both *p* < 0.01). In the hippocampus, in both stressed resilient and stressed resilient DS-treated groups, the expression is significantly higher than in the control group (both *p* < 0.01). The expression in the stressed, resilient, DS-treated group is also significantly increased compared to the untreated, stressed, resilient group and stressed, anhedonic, DS-treated animals (*p* = 0.01, and *p* < 0.01, respectively). To sum up, it has to be noted that untreated, anhedonic mice displayed significant upregulation of *5-Htt*, while in the PFC, no such changes were shown for DS-treated, anhedonic animals. Differential changes between resilient, untreated and DS-treated subgroups were found only for the expression of the *5*-*Ht2A* receptor in the striatum.

### 3.8. Hippocampal Expression of the Plasticity Markers

**Figure 6:**
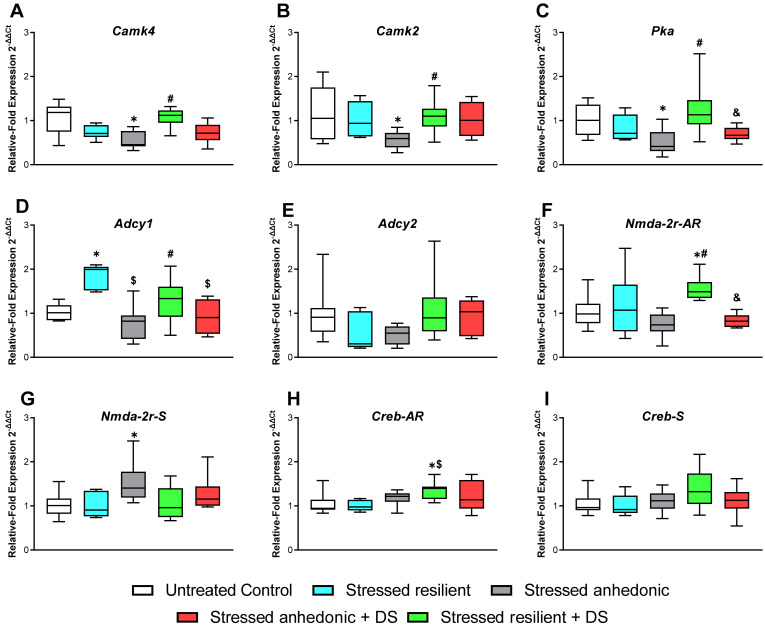
Expression of hippocampal plasticity markers in stressed untreated and DS-treated mice. We found that (**A**) *Camk4* and (**B**) *Camk4* expression were significantly decreased in the untreated, stressed, anhedonic group compared to the control group and the DS-treated, stressed, resilient mice. (**C**) The expression of PKA was significantly decreased in the untreated, stressed, anhedonic group compared to the control group and the DS-treated, stress-resilient mice. The expression in the DS-treated, stressed, anhedonic group was also significantly lower than in the DS-treated, stressed, resilient group, but not compared to the control animals. (**D**) The expression of *Adcy1* was significantly higher in the untreated, stressed, resilient group in comparison to all groups except the DS-treated, stressed, resilient animals. *Adcy1* expression in the DS-treated, stressed, resilient group was significantly increased compared to the untreated, stressed, anhedonic animals. (**E**) There were no significant group differences in *Adcy2* expression. (**F**) *Nmda-2r-AR* expression was significantly increased in the DS-treated, stressed, resilient group compared to all other groups except untreated, stressed, resilient animals. (**G**) *Nmda-2r-S* expression was significantly higher in untreated, stressed, anhedonic mice compared to control animals. (**H**) *Creb-AR* expression in DS-treated, stressed, resilient animals was significantly increased compared to both control and untreated, stressed, resilient animals. (**I**) No significant changes between the groups were observed in *Creb-S* expression. * *p* < 0.05 compared to the control group, # *p* < 0.05 compared to the untreated, anhedonic group, $ *p* < 0.05 compared to the untreated, stressed, resilient group, and & *p* < 0.05 compared to the DS-treated, stressed, resilient group. One-way ANOVA. Data are presented as boxplots with median, first, and third quartiles and minimum to maximum whiskers. Control group *n* = 12, stressed, resilient *n* = 7, stressed, anhedonic *n* = 9, DS-treated, stressed, anhedonic *n* = 10, DS-treated, stressed, resilient *n* = 6.

Given the deficits demonstrated here in the hippocampus-dependent functions, we studied potential changes in the expression of plasticity factors of the brain as possible mechanisms underlying these abnormalities. One-way ANOVA reveals significant alterations in the *Camk4* expression in the hippocampus (F4,38 = 7.1, *p* < 0.01, ordinary one-way ANOVA, Figure 6A). The untreated, stressed, anhedonic group has significantly decreased hippocampal *Camk4* expression compared to the control group (*p* < 0.01, Tukey’s test). The expression of *Camk2* is also significantly altered (W4,16.7 = 6.7, *p* < 0.01, one-way ANOVA, Figure 6B), with a significant decrease in the untreated, stressed, anhedonic mice compared to the control group (*p* = 0.03, Dunnett’s T3 test).

The *Pka* expression is significantly altered (F4,38 = 5.6, *p* < 0.01, ordinary one-way ANOVA, Figure 6C). The *Pka* expression in the untreated, stressed, anhedonic group is significantly decreased compared to the control mice (*p* = 0.03, Tukey’s test), and the expression in the stressed, resilient, DS-treated group is significantly higher than in the stressed, anhedonic, DS-treated group (*p* < 0.05).

The expression of *Adcy1* is significantly affected (F4,38 = 8.9, *p* < 0.01, ordinary one-way ANOVA, Figure 6D). Its expression is significantly increased in the untreated, stressed, resilient group compared to both the control and the untreated, stressed, anhedonic mice (both *p* < 0.01, Tukey’s test). No significant changes are revealed for *Adcy2* hippocampal expression (*p* > 0.05, one-way ANOVA, Figure 5E).

One-way ANOVA reveals significant changes in hippocampal *Nmda-2r-AR* and *Nmda-2r-S* expression (F4,38 = 6.8, *p* < 0.01, and F4,38 = 3.4, *p* = 0.02, respectively, ordinary one-way ANOVA, Figure 6F,G). Groupwise, expression of *Nmda-2r-AR* is significantly increased in stressed, resilient, DS-treated mice in comparison to both the control group and the stressed, anhedonic, DS-treated animals (*p* = 0.01 and *p* < 0.01, respectively, Tukey’s test). The expression of *Nmda-2r-S* is significantly higher in the stressed, anhedonic group than in the controls (*p* = 0.02, Tukey’s test).

Significant alterations are found in *Creb-AR* expression (F4,38 = 6.8, *p* = 0.02, ordinary one-way ANOVA, Figure 6H,I). In the stressed, resilient, DS-treated group, the expression is significantly higher than in the control group and the stressed, resilient group (*p* = 0.02 and *p* = 0.03, respectively, Tukey’s test). No significant differences are found in *Creb-S* hippocampal expression (*p* > 0.05, ordinary one-way ANOVA).

It is of interest that anhedonic, untreated mice displayed a significant decrease in hippocampal expression of key plasticity molecules, such as *Camk4*, *Camk2*, and *Pka*, which was not found in the DS-treated, anhedonic group. The expression of *Nmda-2r-S* was elevated in anhedonic, untreated animals but not in anhedonic, DS-treated mice. DS-treated, resilient mice showed elevated expression of such plasticity factors as *Creb- AR* and *Nmda-2r-AR*, which were not upregulated in untreated, resilient mice, suggesting the role of these molecules in the mechanisms of ameliorated hippocampal functions. Untreated, resilient animals had augmented expression of *Adcy1* that was not found in the DS-treated group, suggesting distinct mechanisms of resilience and a complex interplay of molecular pathways to be induced by applied experimental conditions.

### 3.9. Illumina Study: Mitochondrial Oxidative Phosphorylation Gene Pathway Enrichment

**Figure 7:**
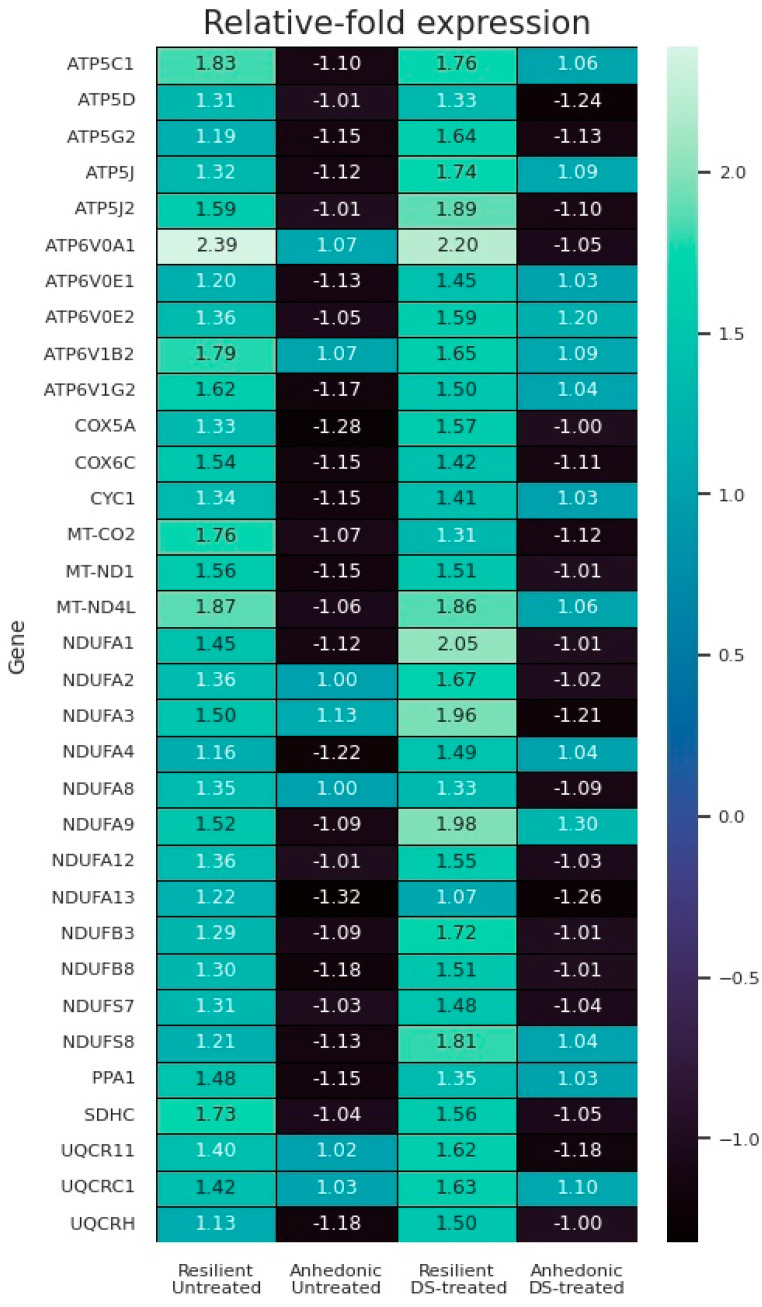
Mitochondrial oxidative phosphorylation pathway enrichment analysis. The expression of mitochondrial NADH dehydrogenase complex was significantly increased in both the resilient, untreated and resilient, DS-treated groups. In both anhedonic, untreated and resilient, DS-treated groups, the expression of this complex was decreased. Similar changes were observed for the expression of succinate dehydrogenase complex, cytochrome bc1 complex, and cytochrome c oxidase complex. The expression of complexes of F-type and V-type ATPase and inorganic pyrophosphatase was significantly increased in resilient, untreated and resilient, DS-treated groups and in DS-treated, anhedonic mice, whereas these measures were significantly decreased in the untreated, anhedonic group.

The results of the pathway enrichment analyses are depicted in Figure 7 and in Figure S1 and Table S1 of Supplementary File. We found that expression of mitochondrial NADH dehydrogenase complex is significantly increased in both resilient, non-treated and resilient, DS groups, while in both anhedonic groups, its expression is decreased. A similar effect is also observed for the expression of succinate dehydrogenase complex, cytochrome bc1 complex, and cytochrome c oxidase complex. Notably, distinctive results have been obtained for the expression of complexes of F-type and V-type ATPase and inorganic pyrophosphatase; while expression of these complexes is increased in both resilient groups, it is also increased in DS-treated, anhedonic mice, as opposed to a decrease in the expression observed in the untreated, anhedonic group. These changes may reflect the decreased efficiency of mitochondrial ATP synthesis in anhedonic mice, which is reversed by DS treatment.

## 4. Discussion

We showed that most of the significant changes in hedonic behaviour, helplessness, nest building, and self-grooming in untreated mice are not observed in the DS-treated mice. In addition, some measures in self-grooming behaviour associated with despair and hippocampus-dependent performance in the Y-maze and marble test are significantly ameliorated in the DS-treated, stressed group compared with the untreated, stressed group. Thus, the behavioural results suggest that applied oral administration of DS counteracts the development of stress-induced MDD-like syndrome in the depression model applied here. However, the decrease in the ratio of anhedonic mice does not reach statistical significance in the stressed, DS-treated group. In addition, DS-treated, stressed mice show ameliorated molecular readouts of neuroinflammation, serotoninergic transmission, and plasticity. Specifically, several molecular features of anhedonic mice, such as overexpression of *Il-1β*, *Tnf*, and *5-Htt* in the PFC, overexpression of *Tnf* and *5-hht* in the raphe dorsalis, and overexpression of *Cox-1* in the hippocampus and striatum, displayed by untreated, anhedonic mice are not found in the DS-treated, anhedonic group. It is noteworthy that the anhedonic groups reveal decreased expression of *5*-*Ht2A* receptor in the hippocampus, while there are markedly opposing changes in the PFC, which also show profound alterations in gene expression. Given that decreased brain 5-*Ht2A* receptor expression and function are well established in patients with MDD [2,94,95], we have evaluated gene expression changes in the hippocampus, assuming that in the employed anhedonic mouse model, molecular mechanisms associated with the effects of DS and individual resilience/susceptibility to MDD-like syndrome are seemingly closely recapitulated in this brain structure.

The DS-treated, anhedonic group demonstrates normalised hippocampal expression of the plasticity molecules *Camk4* and *Camk2*, for which the mRNA levels are decreased in the untreated, anhedonic group. Similarly, while *Nmda-2rs* expression is elevated in the untreated, anhedonic group, this parameter is unchanged in the DS-treated, anhedonic group. The DS-treated, resilient group shows increased *Camk4*, *Pka, Nmda-2r-AR*, and *Creb-AR* expression in the hippocampus compared with the untreated, resilient group, suggesting more pronounced plasticity changes and less distress under the applied treatment. Illumina profiling and pathway analysis reveal marked differences in mitochondrial metabolism of the hippocampus between CMS-vulnerable and CMS-resilient mice, whereas resilient groups reveal an overexpression of mitochondrial complexes, suggesting increased ATP synthesis—that is, activated mitochondrial function. Remarkably, anhedonic mice displayed opposite changes, while the DS-treated, anhedonic group had normalised the expression of F-type and V-type ATPases.

Our results are in line with recent reports suggesting a link between energy metabolism, pro-inflammatory changes, IR-mediated signalling, and synaptic plasticity in MDD [1,20,51,58,96,97] and the role of the hippocampus in these mechanisms [2,94,95]. For example, beneficial therapies in patients with MDD could be related to normalisation of one of the key regulators of energy metabolism, silent information regulator 1 (SIRT1). This protein inhibits the inflammatory response by downregulating the expression of TNF and IL-1β in the CA1 region of the hippocampus, by upregulating the plasticity factor brain-derived neurotrophic factors (BDNF), by normalising the TNF/indoleamine 2,3-dioxygenase (IDO)/5-hydroxytryptamine (5-HT) pathways, and by promoting neurogenesis by activating insulin-like growth factor-1 (IGF-1), an IR agonist [94]. Similarly to that work, we showed that antidepressant-like and memory-enhancing effects of DS treatment are accompanied by its ability to counteract the CMS-induced increase in brain expression of *Tnf, Il-1β*, and *Cox-1*; the CMS-related suppression of *Camk4, Camk2*, and elements of the CREB and 5-HT pathways; and signs of the functional activation of mitochondrial respiratory chain complexes.

We demonstrated that susceptibility but not resilience to stress-induced anhedonia, a core symptom of depression, is associated with significant downregulation of genes encoding mitochondrial respiratory chain complexes, such as NADH dehydrogenase, succinate dehydrogenase, cytochrome *bc*1, cytochrome *c* oxidase, F-type and V-type ATPases, and inorganic pyrophosphatase, suggesting suppression of mitochondrial energy. In contrast, mice resilient to the MDD-like hedonic deficit display a significant decrease in the expression of genes related to these complexes compared with control mice. These data are in line with our earlier findings of a five-fold difference in a number of significantly overexpressed genes in the resilient versus anhedonic mice in the hippocampus [72]. Hence, our study reveals a role for mitochondrial functional activity in the CMS model of depressive-like syndrome.

Keeping with our findings, previous studies have demonstrated that CMS in rodents suppresses the activity of the mitochondrial respiratory chain complexes [98] and alters the mitochondrial ultrastructure [99]. In particular, researchers have found that in rats, 40 days of stress exposure inhibit complex I, III, and IV in the cerebral cortex and cerebellum, while complex II and creatine kinase are not affected [98]. A marked reduction in mitochondrial respiration rates and a dissipated mitochondrial membrane potential in the hippocampus, cortex, and hypothalamus are shown in mice that have been subjected to a variant of the CMS model [99]. These changes are accompanied by increased immobility time in the tail suspension test [99]. In recent work with a chronic restraint stress, bulk transcriptomic analysis on the PFC and nucleus accumbens of mice have uncovered prominent upregulation of mitochondrial DNA (mtDNA) genes encoding oxidative phosphorylation complexes I, III, and IV [58]. Comparison of these data with a transcriptome dataset of subjects with MDD reveals comparable changes in mtDNA-encoded genes in a clinical setting [58]. Thus, pre-clinical studies suggest that changes in mitochondrial gene expression can be important players in susceptibility to MDD. However, this question has been not addressed experimentally by comparing vulnerable versus resilient-to-stress-induced-anhedonia cohorts of animals.

Interestingly, mitochondrial functional downregulation in the brain is inter-connected with altered microglial glucose metabolism [100]. In the model we employ here, microglial activation has been reported as a specific feature of anhedonia-vulnerable but not anhedonia-resilient mice, as shown for the hippocampus [57] and the PFC [78]. The differences in the numbers of Iba-1-positive cells between the subgroups are accompanied by overexpression of COX-2 in neurons in the CA1 area and the dentate gyrus of the hippocampus, as well as signs of suppressed neurogenesis (for example, a reduction in Ki67-positive cells in the subgranular zone of the hippocampus in mice susceptible to anhedonia) [57]. The latter differences are in line with the differences we describe in the expression of plasticity molecules between the anhedonic and resilient groups, where the former display a significant decrease in the hippocampal expression of *Camk4, Camk2, Pka, Adcy1* and *Creb-AR*. We report opposing changes for mice resilient to anhedonia. Notably, compared with untreated animals, DS-treated resilient and anhedonic animals have higher or ‘rescued’ mRNA levels of these plasticity-related molecules in the hippocampus. These differences could explain the unaltered performance of resilient mice in the hippocampus-dependent tasks in our study.

Our results are consistent with current views on the adaptive role of hippocampal plasticity factors during stress. For example, CREB is one of the most studied plasticity molecules. It plays a pivotal role in convergence among several molecular pathways and controls the transcription of stress-sensitive genes that regulate responses to rewarding and stressful stimuli in a brain-region-specific manner, as well as stress susceptibility [97,101]. Important roles in the mechanisms of plasticity and adaptation have also been shown for other plasticity-related molecules for which the expression changes in the hippocampus of resilient and anhedonic mice, such as adenylyl cyclase [102,103], PKA [82], CAMK2, and CAMK4 [82,104]. In parallel to these changes, we have found differential expression of NMDA-receptor-signalling-related molecules between the groups. These proteins are known to be related to the expression of hippocampal plasticity factors during stress [82,104,105].

The present study suggests the suppression of mitochondrial metabolism in the hippocampus of stressed, anhedonic mice and the activation of metabolic functions in mice resilient to stress-induced anhedonia. These changes are accompanied by increased gene expression of pro-inflammatory molecules across various brain structures and decreased hippocampal expression of plasticity factors in the anhedonic, but not the resilient, mice. The changes in anhedonic mice are less pronounced under conditions of pharmacologically enhanced IR-mediated signalling via chronic administration of DS. This amelioration through the administration of IRS, as applied here, is accompanied by partially normalised emotional behaviours of stressed mice.

Our findings indicate a relationship between the pharmacological stimulation of IR with DS and changes in the expression of mitochondrial respiratory chain genes in stressed animals that differ in their vulnerability to stress-induced anhedonia. In general, these results further support the view that mitochondrial energy regulation contributes to an individual’s predisposition to MDD [52,58]. The normalising effects of DS on these outcomes, together with our earlier reports showing the effects of DS on depressive-like behaviours [45,46,47,48] and data linking the susceptibility of mice to CMS-induced anhedonia with lowered brain activities of catalase and superoxide dismutase [47], suggest that reduced mitochondrial energy levels may mediate the effects of stress or other factors on individual predisposition to anhedonia development. As such, the beneficial effects of IRSs or other remedies that elevate mitochondrial respiration can alleviate depressive features and deficits in hippocampal functions.

Previous studies with the CMS model, which stratify mice based on their susceptibility to stress-induced anhedonia, have demonstrated that it can be related to changes in the expression of various markers of inflammation that are not shown in rodents resilient to anhedonia [53,57,78,106,107]. For instance, CMS-exposed mice susceptible to anhedonia have shown elevations of *Cox-2* expression in the hippocampus and *Cox-1* and *Ido* expression in the midbrain raphe region [77,78]. This suggests a possible interaction of neuroinflammation with altered 5-HT transmission–related mechanisms in anhedonic mice [77,78]. The latter group of animals, but not resilient mice, show overexpression of TNF mRNA and an increased number of Iba-1-positive cells in the PFC [78] and in the hippocampus [57]. The functional effects of elevated IL-1β levels in the CNS in anhedonic mice, but not resilient mice, are associated with stress and susceptibility to MDD in patients.

While the limitation of our study is the use of only data from one time point in the analysis of gene expression changes that, in stressed mice, can potentially be affected by MDD-like alterations in circadian rhythmicity [47,78,108], in a context of individual resilience to this syndrome and the impact of mitochondrial functions, our data are in line with similar findings [1,2,3,4,5].

Our study reveals the antidepressant-like activity of oral use of DS, suggesting that this or similar IRSs might be exploited to treat MDD. Indeed, addressing insufficient mitochondrial functions and energy balance with compounds, such as DS, would represent a move towards more advanced, personalised treatment of depression. This is further demonstrated in epidemiological studies that have revealed a bi-directional relationship between incidence of MDD, on one side, and medical manifestations of insulin resistance, on the other side [20].

More than a decade ago, the World Health Organization identified MDD as a ‘global crisis’ [109]. The COVID-19 pandemic has aggravated the situation [110,111]. Despite the variety of therapeutics used for patients with depression, how to manage this disease within society remains largely unsolved [109]. Hence, the development of new, more effective antidepressant remedies is an ongoing need in psychopharmacology, as the growing prevalence of MDD markedly diminishes quality of life and greatly affects the medical and socioeconomic situation within society [112]. Up until now, the common treatment for this disorder remains monotherapy with classic antidepressants—that is, targeting predominantly monoaminergic neurotransmission [113,114]—but their use is not sufficiently effective in all patients [115]. In this context, the use of IRSs might be of particular value owing to the growing experience with these drugs among other drug candidates and novel compounds with antidepressant activities [3,27].

## 5. Conclusions

Collectively, our work reinforces the idea that changes in mitochondrial gene expression are key players in the brain adaptations associated with an individual’s susceptibility and resilience to MDD. The evidence suggests that drugs, such as IRSs, that increase IR-mediated signaling can be used as an adjunctive therapy for depression.

## Data Availability

Data are available upon request.

## Supplementary Materials

The following supporting information can be downloaded at: https://www.mdpi.com/article/10.3390/biom13121782/s1, Figure S1: Number of resilient and anhedonic animals in untreated and DS-treated groups; Figure S2: Comparison of behavioural parameters of stressed anhedonic and resilient animals; Table S1: Expression of mitochondrial oxidative phosphorylation gene pathways; Table S2: Summary of comparisons in the measures of inflammatory markers and 5-HT-related genes expression changes across various brain regions of stressed untreated and DS-treated mice.
