# Supplementary material for: Resilience and Vulnerability to Stress-Induced Anhedonia: Unveiling Brain Gene Expression and Mitochondrial Dynamics in a Mouse Chronic Stress Depression Model
Source: Biomolecules. 2023 Dec 12;13(12):1782. doi: 10.3390/biom13121782 (PMC10741640; doi:10.3390/biom13121782)
Supplement: Supplementary file 1 [file biomolecules-13-01782-s001.zip › biomolecules-2728980-supplementary.pdf]

Supplementary File

Results

Fisher's exact test did not reveal significant differences in the number of anhedonic animals in untreated and DS-treated groups (p=0.29, Fisher's exact test, Fig. S1).

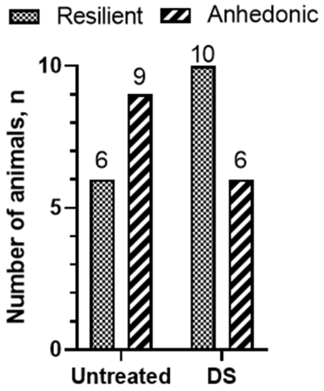

Figure S1. Number of resilient and anhedonic animals in untreated and DS-treated groups.

Behavioural comparison between stressed untreated and DS-treated resilient and anhedonic groups

Tail suspension test

In the duration of immobility in the tail suspension test, two-way ANOVA revealed a significant main effect of anhedonia factor ($F_{1,27}$=23.17, p<0.01, two-way ANOVA, Fig. S2A), but not of treatment factor or the interaction ($F_{1,27}$=0.02, p=0.88, and $F_{1,27}$=1.43, p=0.24., respectively, two-way ANOVA). Post-hoc test revealed a significant increase of the immobility duration in both anhedonic groups compared to treatment-matched resilient groups (untreated: p=0.04; DS-treated: p<0.01, Šídák's test).

In the latency to the first immobility episode, both main factors of anhedonia and treatment were significant ($F_{1,27}$=20.70, p<0.01, and $F_{1,27}$=9.96, p<0.01, respectively two-way ANOVA Fig. S2B). No significant interaction of the factors was found ($F_{1,27}$=0.0002, p=0.99, two-way ANOVA). Group difference was observed between treatment-matched groups: in both untreated and DS-treated groups, latency to immobility was significantly decreased in the anhedonic mice (both p=0.02, Tukey's test). In the DS-treated resilient group, this parameter was significantly higher than in untreated anhedonic group (p=0.02, Tukey's test) We found no group differences between untreated and DS-treated subgroups with

respect to the development of anhedonia in the latency to the first immobility episode (resilient: $p=0.14$, anhedonic $p=0.14$, Tukey's test).

Splash test

In the splash test, latency to groom was significantly affected by both anhedonia and treatment main factors ($F_{1,27}=67.49$, $p<0.01$, and $F_{1,27}=4.38$, $p<0.05$, respectively, two-way ANOVA, Fig. S2C). No significant interaction was found ($F_{1,27}=1.39$, $p=0.26$, two-way ANOVA). In both untreated and DS-treated mice, latency to groom was significantly higher in anhedonic animals compared to both resilient groups (all $p<0.01$, Tukey's test), and to. No group differences were found between untreated and DS-treated subgroups with respect to the development of anhedonia in the latency top groom (resilient: $p=0.9$, anhedonic $p=0.13$, Tukey's test).

Duration of grooming in the splash test was significantly affected by the interaction of anhedonia and treatment factors ($F_{1,27}=5.53$, $p=0.03$, two-way ANOVA, Fig. S2D). Groupwise, in both untreated and DS-treated anhedonic mice, duration of grooming was significantly decreased compared to both resilient groups (all $p<0.05$, Tukey's test). No group differences were found between untreated and DS-treated subgroups with respect to this parameter (resilient: $p=0.07$, anhedonic $p=0.88$, Tukey's test).

In number of grooming episodes, both anhedonia and treatment main factors had significant effect ($F_{1,27}=12.33$, $p<0.01$, and $F_{1,27}=4.68$, $p=0.04$, two-way ANOVA, Fig. S2E), but not their interaction ($F_{1,27}=3.06$, $p=0.09$, two-way ANOVA). Post-hoc test revealed in the DS-treated resilient group, number of grooming episodes was significantly higher than in both DS-treated and untreated anhedonic groups (both $p<0.01$, Tukey's test) and untreated resilient mice ($p=0.04$, Tukey's test). No group differences were found between untreated and DS-treated anhedonic subgroups with respect to number of grooming episodes ($p=0.99$, Tukey's test).

Pellet displacement test

Only the main factor of anhedonia was significant for the latency to displace first food pellet in the pellet displacement test ($F_{1,27}=24.45$, $p<0.01$, two-way ANOVA, Fig. S2F). The interaction was borderline significant ($F_{1,27}=4.21$, $p=0.050$, two-way ANOVA), and no significant effect of treatment main factor was found ($F_{1,27}=2.07$, $p=0.16$, two-way ANOVA). In the untreated anhedonic group, the latency to

displace was significantly increased compared to the untreated resilient group ($p<0.01$, Šídák's test), while no significant difference was found between DS-treated groups ($p=0.09$, Šídák's test).

The number of food pellets displaced during the first 20 minutes of the test was significantly affected by the anhedonia factor ($F_{1,27}=30.03$, $p<0.01$, two-way ANOVA, Fig.S2G), but not by the treatment factor or their interaction ($F_{1,27}=3.18$, $p=0.09$, and $F_{1,27}=1.25$, $p=0.27$, respectively, two-way ANOVA). In both anhedonic groups, number of the pellets displaced in first 20 minutes of the test was significantly lower compared to treatment-matched resilient groups (untreated: $p=0.01$, DS-treated: $p<0.01$, Šídák's test).

Coat disintegration and nest building

Kruskal-Wallis test revealed a significant difference in the coat disintegration assessment ($p<0.01$, Kruskal-Wallis test, fig. S2H). In the untreated groups, coat score of the anhedonic mice was significantly lower than in resilient animals ($p=0.03$, Dunn's test). No group differences were found between untreated and DS-treated subgroups (anhedonic: $p=0.82$, resilient $p>0.99$, Dunn's test).

Significant differences were found by Kruskal-Wallis test in the nest building score ($p<0.01$, Kruskal-Wallis test, Fig. S2I), however no group differences were found by post-hoc Dunn's test.

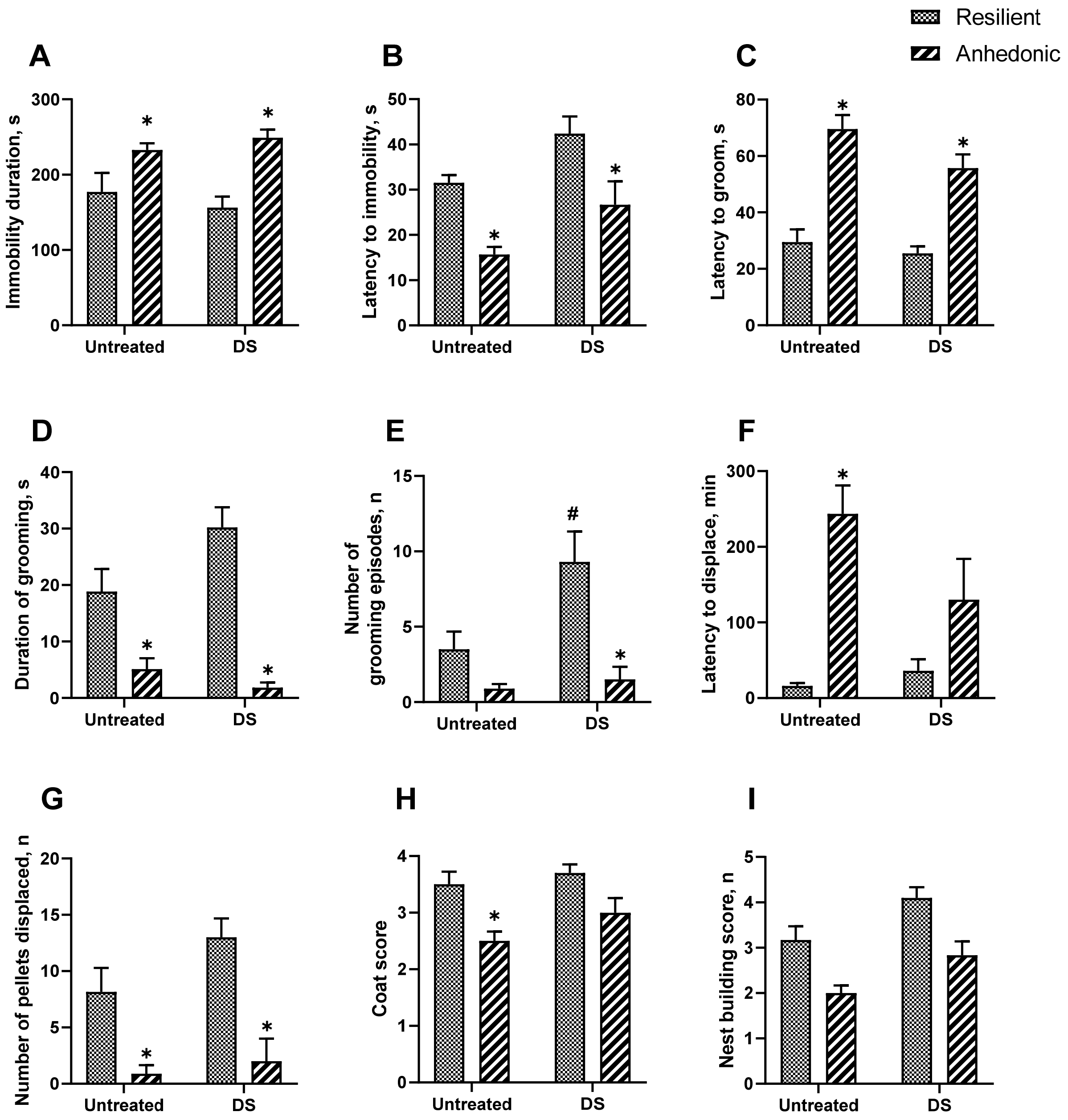

Figure S2. Comparison of behavioural parameters of stressed anhedonic and resilient animals. In the tail suspension test, in comparison with treatment-matched resilient groups ,both anhedonic groups showed **(A)** significantly increased immobility duration and **(B)** decreased latency to immobility. In the splash test, **(C)** significantly increased latency to groom, and **(D)** significantly reduced duration of grooming in the anhedonic animals compared to treatment-matched resilient mice was found, **(E)** the number of grooming episodes was significantly elevated in DS-treated resilient mice compared to both anhedonic DS-treated mice and resilient untreated mice. In the pellet displacement test, **(F)** the latency to displace the first pellet was significantly increased in untreated anhedonic group compared to the untreated resilient group, while no significant difference was observed between the DS-treated stressed subgroups; **(G)** both anhedonic subgroups displayed a significantly less pellets displaced during the first 20 minutes of the test than respective resilient groups of mice. Coat disintegration score **(H)** and nest building score **(I)** were significantly lowered in both anhedonic groups as compared to treatment-matched resilient groups. *$p<0.05$ vs. treatment-matched resilient group, #$p<0.05$ vs. untreated resilient group, two-way ANOVA with Tukey's test or Šídák's test. Data is presented as mean±SEM.

Table S1. Expression of mitochondrial oxidative phosphorylation gene pathways.

Gene Symbol	Entrez Gene Name	Expression fold-change				Entrez Gene ID	
		Resilient Untreated	Anhedonic Untreated	Resilient DS-treated	Anhedonic DS-treated	Human	Mouse
ATP5C1	ATP synthase, H+ transporting, mitochondrial F1 complex, gamma polypeptide 1	1.834	−1.097	1.764	1.058	509	11949
ATP5D	ATP synthase, H+ transporting, mitochondrial F1 complex, delta subunit	1.307	−1.008	1.327	−1.236	513	66043
ATP5G2	ATP synthase, H+ transporting, mitochondrial Fo complex, subunit C2 (subunit 9)	1.19	−1.146	1.644	−1.129	517	67942
ATP5J	ATP synthase, H+ transporting, mitochondrial Fo complex, subunit F6	1.321	−1.123	1.739	1.089	522	11957
ATP5J2	ATP synthase, H+ transporting, mitochondrial Fo complex, subunit F2	1.588	−1.006	1.887	−1.104	9551	57423
ATP6V0A1	ATPase, H+ transporting, lysosomal V0 subunit a1	2.389	1.072	2.201	−1.055	535	11975
ATP6V0E1	ATPase, H+ transporting, lysosomal 9kDa, V0 subunit e1	1.203	−1.128	1.446	1.035	8992	11974
ATP6V0E2	ATPase, H+ transporting V0 subunit e2	1.357	−1.05	1.592	1.203	155066	76252
ATP6V1B2	ATPase, H+ transporting, lysosomal 56/58kDa, V1 subunit B2	1.792	1.067	1.648	1.094	526	11966
ATP6V1G2	ATPase, H+ transporting, lysosomal 13kDa, V1 subunit G2	1.618	−1.171	1.504	1.044	534	66237
COX5A	cytochrome c oxidase subunit Va	1.328	−1.278	1.569	−1.005	9377	12858
COX6C	cytochrome c oxidase subunit VIc	1.541	−1.154	1.422	−1.114	1345	12864
CYC1	cytochrome c-1	1.343	−1.151	1.409	1.026	1537	66445
MT-CO2	cytochrome c oxidase subunit II	1.759	−1.068	1.307	−1.124	4513	17709
MT-ND1	NADH dehydrogenase, subunit 1 (complex I)	1.559	−1.153	1.506	−1.012	4535	17716
MT-ND4L	NADH dehydrogenase, subunit 4L (complex I)	1.869	−1.059	1.865	1.057	4539	17720
NDUFA1	NADH dehydrogenase (ubiquinone) 1 alpha subcomplex, 1, 7.5kDa	1.451	−1.117	2.048	−1.008	4694	54405
NDUFA2	NADH dehydrogenase (ubiquinone) 1 alpha subcomplex, 2, 8kDa	1.36	1.003	1.666	−1.02	4695	17991
NDUFA3	NADH dehydrogenase (ubiquinone) 1 alpha subcomplex, 3, 9kDa	1.498	1.128	1.956	−1.21	4696	66091
NDUFA4	NADH dehydrogenase (ubiquinone) 1 alpha subcomplex, 4, 9kDa	1.157	−1.218	1.485	1.037	4697	17992
NDUFA8	NADH dehydrogenase (ubiquinone) 1 alpha subcomplex, 8, 19kDa	1.348	1.002	1.329	−1.093	4702	68375
NDUFA9	NADH dehydrogenase (ubiquinone) 1 alpha subcomplex, 9, 39kDa	1.516	−1.093	1.983	1.297	4704	66108
NDUFA12	NADH dehydrogenase (ubiquinone) 1 alpha subcomplex, 12	1.365	−1.009	1.55	−1.035	55967	66414
NDUFA13	NADH dehydrogenase (ubiquinone) 1 alpha subcomplex, 13	1.223	−1.322	1.072	−1.264	51079	67184
NDUFB3	NADH dehydrogenase (ubiquinone) 1 beta subcomplex, 3, 12kDa	1.295	−1.088	1.717	−1.014	4709	66495

NDUFB8	NADH dehydrogenase (ubiquinone) 1 beta subcomplex, 8, 19kDa	1.303	−1.178	1.513	−1.012	4714	67264
NDUFS7	NADH dehydrogenase (ubiquinone) Fe-S protein 7, 20kDa (NADH-coenzyme Q reductase)	1.314	−1.026	1.479	−1.041	374291	75406
NDUFS8	NADH dehydrogenase (ubiquinone) Fe-S protein 8, 23kDa (NADH-coenzyme Q reductase)	1.211	−1.134	1.807	1.044	4728	225887
PPA1	pyrophosphatase (inorganic) 1	1.483	−1.149	1.355	1.027	5464	67895
SDHC	succinate dehydrogenase complex, subunit C, integral membrane protein, 15kDa	1.734	−1.042	1.56	−1.054	6391	66052
UQCR11	ubiquinol-cytochrome c reductase, complex III subunit XI	1.399	1.02	1.624	−1.177	10975	66594
UQCRC1	ubiquinol-cytochrome c reductase core protein I	1.415	1.031	1.627	1.1	7384	22273
UQCRH	ubiquinol-cytochrome c reductase hinge protein	1.13	−1.175	1.502	−1.004	7388	100042918

There were opposite changes in the expression of mitochondrial oxidative phosphorylation pathways in the hippocampus of anhedonic vs. treatment-matched resilient mice, normalized to values of non-stressed untreated controls. Resilient to stress-induced anhedonia mice showed overall an upregulation. Anhedonic mice revealed a general decrease in mitochondrial oxidative phosphorylation pathways that was partially ameliorated in DS-treated animals. Genes symbols and names for Entrez PubMed, human and mouse IDs are indicated (see also ms text).

Table S2. Summary of comparisons in the measures of inflammatory markers and 5-HT-related genes expression changes across various brain regions of stressed untreated and DS-treated mice (one-way ANOVA, Tukey's test, *p<0.05). *For detailed description of the results, see section 3.7 and Fig. 5 of the manuscript.*

Il-1β **mRNA expression**

**Prefrontal cortex**				
One-way ANOVA	**F(4, 25) = 5.11**		**p = 0.0038***	
Tukey's test p-value vs.	Untreated Control	Stressed Resilient	Stressed Anhedonic	Stressed Resilient + DS
Stressed Resilient	0.6568	—		
Stressed Anhedonic	**0.0040***	0.0910	—	
Stressed Resilient + DS	0.1397	0.8195	0.5230	—
Stressed Anhedonic + DS	**0.0194***	0.2972	0.9639	0.8847
**Hippocampus**				
One-way ANOVA	**F(4, 25) = 6,977**		**p = 0.0006***	
Tukey's test p-value vs.	Untreated Control	Stressed Resilient	Stressed Anhedonic	Stressed Resilient + DS
Stressed Resilient	**0.0367***	—		
Stressed Anhedonic	**0.0005***	0.4131	—	
Stressed Resilient + DS	0.5019	0.5955	**0.0260***	—
Stressed Anhedonic + DS	**0.0115***	0.9865	0.7130	0.3123
**Raphe**				
One-way ANOVA	**F(4, 25) = 8.152**		**p = 0.0002***	
Tukey's test p-value vs.	Untreated Control	Stressed Resilient	Stressed Anhedonic	Stressed Resilient + DS
Stressed Resilient	0.0538	—		
Stressed Anhedonic	**<0.0001***	0.1048	—	
Stressed Resilient + DS	0.0975	0.9984	0.0581	—
Stressed Anhedonic + DS	**0.0042***	0.8122	0.5748	0.6504
**Striatum**				
One-way ANOVA	**F(4, 25) = 8.730**		**p = 0.0001***	
Tukey's test p-value vs.	Untreated Control	Stressed Resilient	Stressed Anhedonic	Stressed Resilient + DS
Stressed Resilient	0.0576	—		
Stressed Anhedonic	**0.0003***	0.2430	—	
Stressed Resilient + DS	0.4511	0.7680	**0.0227***	—
Stressed Anhedonic + DS	**0.0007***	0.3789	0.9984	**0.0435***
**Motor cortex**				
One-way ANOVA	**F(4, 25) = 2.805**		**p = 0.0473***	
Tukey's test p-value vs.	Untreated Control	Stressed Resilient	Stressed Anhedonic	Stressed Resilient + DS
Stressed Resilient	0.9129	—		
Stressed Anhedonic	0.0724	0.3356	—	
Stressed Resilient + DS	0.8292	0.9996	0.4451	—
Stressed Anhedonic + DS	0.1063	0.4373	0.9997	0.5582

Table S2. ***Continued***

Tnf **mRNA expression**

**Prefrontal cortex**				
One-way ANOVA	**F(4, 25) = 4.957**		**p = 0.0044***	
Tukey's test p-value vs.	Untreated Control	Stressed Resilient	Stressed Anhedonic	Stressed Resilient + DS
Stressed Resilient	0.9993	—		
Stressed Anhedonic	**0.0109***	**0.0062***	—	
Stressed Resilient + DS	0.9872	0.9485	**0.0342***	—
Stressed Anhedonic + DS	0.4841	0.3549	0.3147	0.7779

**Hippocampus**				
One-way ANOVA	**F(4, 25) = 5.627**		**p = 0.0023***	
Tukey's test p-value vs.	Untreated Control	Stressed Resilient	Stressed Anhedonic	Stressed Resilient + DS
Stressed Resilient	0.6054	—		
Stressed Anhedonic	**0.0018***	0.0569	—	
Stressed Resilient + DS	0.9837	0.8872	**0.0068***	—
Stressed Anhedonic + DS	0.3186	0.9862	0.1562	0.6196

**Raphe**				
One-way ANOVA	F(4, 25) = 1.763		p = 0.1693	
	No significant differences			

**Striatum**				
One-way ANOVA	F(4, 25) = 1.366		p = 0.2738	
	No significant differences			

**Motor cortex**				
One-way ANOVA	F(4, 25) = 2.188		p = 0.0995	
	No significant differences			

Cox-1 **mRNA expression**

**Prefrontal cortex**				
One-way ANOVA	**F(4, 25) = 14.10**		**p < 0.0001***	
Tukey's test p-value vs.	Untreated Control	Stressed Resilient	Stressed Anhedonic	Stressed Resilient + DS
Stressed Resilient	**<0.0001***	—		
Stressed Anhedonic	**<0.0001***	>0.9999	—	
Stressed Resilient + DS	**0.0007***	0.4832	0.4132	—
Stressed Anhedonic + DS	**0.0001***	0.8734	0.8179	0.9559

**Hippocampus**				
One-way ANOVA	**F(4, 25) = 5.064**		**p = 0.004***	
Tukey's test p-value vs.	Untreated Control	Stressed Resilient	Stressed Anhedonic	Stressed Resilient + DS
Stressed Resilient	0.0839	—		
Stressed Anhedonic	**0.0034***	0.6446	—	
Stressed Resilient + DS	0.7821	0.5429	0.0509	—
Stressed Anhedonic + DS	0.8078	0.5129	**0.0457***	>0.9999

Table S2. ***Continued***

Raphe

One-way ANOVA	**F(4, 25) = 3.824**		**p = 0.0246***	
Tukey's test p-value vs.	Untreated Control	Stressed Resilient	Stressed Anhedonic	Stressed Resilient + DS
Stressed Resilient	0.2315	—		
Stressed Anhedonic	**0.0225***	0.6905	—	
Stressed Resilient + DS	0.0503	0.8988	0.9924	—
Stressed Anhedonic + DS	0.5328	0.9687	0.3428	0.5733

Striatum

One-way ANOVA	**F(4, 25) = 3.052**		**p = 0.0354***	
Tukey's test p-value vs.	Untreated Control	Stressed Resilient	Stressed Anhedonic	Stressed Resilient + DS
Stressed Resilient	0.2461	—		
Stressed Anhedonic	**0.0229***	0.7664	—	
Stressed Resilient + DS	0.0892	0.9812	0.9697	—
Stressed Anhedonic + DS	0.2652	>0.9999	0.7409	0.9748

Motor cortex

One-way ANOVA	**F(4, 25) = 2.936**		**p = 0.0406***	
Tukey's test p-value vs.	Untreated Control	Stressed Resilient	Stressed Anhedonic	Stressed Resilient + DS
Stressed Resilient	0.5588	—		
Stressed Anhedonic	0.0526	0.6380	—	
Stressed Resilient + DS	0.0620	0.6849	>0.9999	—
Stressed Anhedonic + DS	0.1580	0.9139	0.9812	0.9894

***5-Htt* mRNA expression**

Prefrontal cortex

One-way ANOVA	**F(4, 25) = 11.70**		**p < 0.0001***	
Tukey's test p-value vs.	Untreated Control	Stressed Resilient	Stressed Anhedonic	Stressed Resilient + DS
Stressed Resilient	0.9873	—		
Stressed Anhedonic	**<0.0001***	**0.0001***	—	
Stressed Resilient + DS	0.9940	>0.9999	**0.0001***	—
Stressed Anhedonic + DS	0.6944	0.9264	**0.0010***	0.8967

Hippocampus

One-way ANOVA	**W(4, 11.56) = 3.073**		**p = 0.0266***	
Tukey's test p-value vs.	Untreated Control	Stressed Resilient	Stressed Anhedonic	Stressed Resilient + DS
Stressed Resilient	0.3352	—		
Stressed Anhedonic	0.5620	0.6356	—	
Stressed Resilient + DS	>0.9999	0.3896	0.5666	—
Stressed Anhedonic + DS	0.1300	0.8208	0.7098	0.1435

Raphe

One-way ANOVA	F(4, 25) = 0.8388	p = 0.5136
	No significant differences	

Table S2. ***Continued***

Striatum

One-way ANOVA	F(4, 25) = 2.039		p = 0.1196	
	No significant differences			

Motor cortex

One-way ANOVA	**F(4, 10.23) = 3.738**		**p = 0.0403***	
Tukey's test p-value vs.	Untreated Control	Stressed Resilient	Stressed Anhedonic	Stressed Resilient + DS
Stressed Resilient	0.9943	—		
Stressed Anhedonic	0.2332	0.2952	—	
Stressed Resilient + DS	0.9932	>0.9999	0.2902	—
Stressed Anhedonic + DS	0.9469	0.9982	0.3510	0.9978

***5Htr2a* mRNA expression**

Prefrontal cortex

One-way ANOVA	**F(4, 25) = 12.32**		**p < 0.0001***	
Tukey's test p-value vs.	Untreated Control	Stressed Resilient	Stressed Anhedonic	Stressed Resilient + DS
Stressed Resilient	0.3213	—		
Stressed Anhedonic	**0.0001***	**0.0186***	—	
Stressed Resilient + DS	0.9833	0.6249	**0.0006***	—
Stressed Anhedonic + DS	**0.0003***	**0.0383***	0.9977	**0.0013***

Hippocampus

One-way ANOVA	**F(4, 25) = 18.60**		**p < 0.0001***	
Tukey's test p-value vs.	Untreated Control	Stressed Resilient	Stressed Anhedonic	Stressed Resilient + DS
Stressed Resilient	**0.0093***	—		
Stressed Anhedonic	0.5620	0.2311	—	
Stressed Resilient + DS	**<0.0001***	**`0.0108***	**<0.0001***	—
Stressed Anhedonic + DS	>0.9999	**0.0114***	0.6132	**<0.0001***

Raphe

One-way ANOVA	F(4, 25) = 1.948		p = 0.1336	
	No significant differences			

Striatum

One-way ANOVA	**F(4, 25) = 5.649**		**p = 0.0022**	
Tukey's test p-value vs.	Untreated Control	Stressed Resilient	Stressed Anhedonic	Stressed Resilient + DS
Stressed Resilient	0.0820	—		
Stressed Anhedonic	**0.0023***	0.5495	—	
Stressed Resilient + DS	**0.0360***	0.9951	0.7770	—
Stressed Anhedonic + DS	**0.0048***	0.7314	0.9981	0.9115

Motor cortex

One-way ANOVA	F(4, 25) = 0.4424		p = 0.7768	
	No significant differences			
